# Indole Alkaloids of the Stigonematales (Cyanophyta): Chemical Diversity, Biosynthesis and Biological Activity

**DOI:** 10.3390/md14040073

**Published:** 2016-04-06

**Authors:** Katherine Walton, John P. Berry

**Affiliations:** Department of Chemistry and Biochemistry, Marine Science Program, Florida International University, 3000 NE 151st Street, North Miami, FL 33181, USA; kwalt001@fiu.edu

**Keywords:** cyanobacteria, blue-green algae, Stigonematales, indole alkaloids, hapalindole, ambiguine, fischerindole, welwitindolinone, toxins, harmful algal blooms

## Abstract

The cyanobacteria are well recognized as producers of a wide array of bioactive metabolites including toxins, and potential drug candidates. However, a limited number of taxa are generally considered with respect to both of these aspects. That said, the order Stigonematales, although largely overlooked in this regard, has become increasingly recognized as a source of bioactive metabolites relevant to both human and environmental health. In particular, the hapalindoles and related indole alkaloids (*i.e.*, ambiguines, fischerindoles, welwitindolinones) from the order, represent a diverse, and phylogenetically characteristic, class of secondary metabolites with biological activity suggestive of potential as both environmental toxins, and promising drug discovery leads. The present review gives an overview of the chemical diversity of biologically active metabolites from the Stigonematales—and particularly the so-called *hapalindole-type alkaloids*—including their biosynthetic origins, and their pharmacologically and toxicologically relevant bioactivities. Taken together, the current evidence suggests that these alkaloids, and the associated cyanobacterial taxa from the order, warrant future consideration as both potentially harmful (*i.e.*, “toxic”) algae, and as promising leads for drug discovery.

## 1. Introduction

Cyanobacteria are ubiquitous in the environment, but arguably best recognized as the conspicuous “blue-green algae” in freshwater and coastal habitats, and particularly in association with eutrophication and episodic “harmful algal blooms” (HABs). This is reinforced by the recognized diversity of toxic, and more generally bioactive, metabolites by this phylum. Studies of the cyanobacteria have, in particular, focused on the role of several metabolites—the so-called *cyanotoxins*—with documented toxicity (e.g., human and livestock poisonings, chronic health effects, *etc.*). In parallel, pharmacological investigations have sought to leverage this chemical diversity in regards to possible drug discovery leads. The chemical diversity of cyanobacteria with respect to both areas is more generally reviewed elsewhere [1,2,3,4,5].

Among studies of bioactive metabolites from cyanobacteria, the order Stigonematales remains relatively underrepresented with regards to both environmental health (*i.e.*, toxin) and biomedical (*i.e.*, drug discovery) aspects. Lack of investigation, with respect to the former, arguably stems from the focus on a limited number of recognized toxic metabolites with established links to HAB events, or human health concerns (e.g., contamination of drinking water), and the thus taxa (*i.e.*, Chroococcales, Nostocales, Oscillatoriales) which produce these compounds. Specifically, investigations of the toxic potential of cyanobacteria have consequently focused on well-described neurotoxins (e.g., saxitoxin, anatoxin-a, BMAA) and “hepatotoxic” metabolites (e.g., microcystins, nodularin, cylindrospermopsin), and therefore, the taxa most frequently associated with these compounds (e.g., *Microcystis*, *Nodularia*, *Cylindrospermopsis*, *Anabaena*, *Aphanizomenon*, *Lyngbya)* [3,5]. Similarly, the vast majority of investigations of cyanobacterial metabolites explored with respect to potential drug leads have been, likewise, largely biased toward—and presumptively due to the tremendous chemical diversity of—a rather small taxonomic subset of cyanobacteria, and most notably the polyphyletic genus, *Lyngbya* (Order Oscillatoriales), and limited number of other genera (e.g., *Nostoc*, *Symploca*) [1,2,4].

That said, an accumulated knowledge of the chemistry of the Stigonematales, from more than three decades of investigation, suggests that the order represents a unique repertoire of bioactive metabolites with relevance to both drug discovery and environmental health. In particular, a chemically diverse family of bioactive indole alkaloids is seemingly characteristic and, in fact, phylogenetically restricted to the order. Herein, we specifically review this family of unique indole alkaloids, and to a lesser extent other bioactive metabolites, from the Stigonematales with respect to their chemical diversity—including biosynthesis and pharmacology/toxicology—specifically as it relates to their potential as drug leads, possible ecological function and relevance to environmental health.

## 2. Biology of the Stigonematales

**Figure 1:**
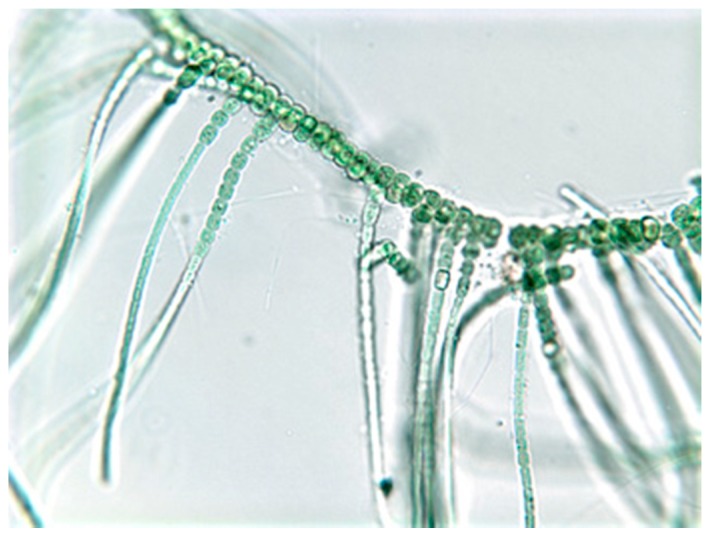
Photomicrograph of *Fischerella* 52-1 as a representative of the branched filamentous habit of the Stigonematales.

Members of the Stigonematales are classified as heterocystous (*i.e.*, nitrogen-fixing) cyanobacteria, and within this classification, specifically distinguished as “true branching” filamentous cyanobacteria (Figure 1). Heterocysts form within filaments, particularly during nitrogen starvation, and are able to enzymatically (via nitrogenases) utilize dinitrogen (*i.e.*, N_2_). Taxa which produce heterocysts have been shown [6] to represent a monophyletic group (based on 16s rDNA) within the cyanobacteria previously referred to as the orders Nostocales and Stigonematales, but classified by more recent taxonomic nomenclature as Section IV and V, respectively. That said, the Stigonematales have been shown, in fact, to represent a polyphyletic group sub-classified as “T-branching” and “Y-branching” groups [7,8]. In the current review, however, we will employ the classic nomenclature of Order Stigonematales.

In addition to being heterocystous, the Stigonematalean cyanobacteria are also capable of producing hormogonia (motile trichomes) and akinetes (thick-walled dormant cells) adding, thereby, to their ecological success. In fact, the Stigonematales represent the most complex prokaryotes, and were originally thought to be directly ancestral to eukaryotes [9]. Moreover, the order represents an ecologically diverse group with representatives isolated from a wide range of terrestrial and freshwater habitats, including numerous thermophiles, as well as notable epiphytic species.

With respect to bioactive metabolites, a relatively limited number of taxa within the order, however, have been investigated. And, to-date, only six genera representing only three (of the eight described) families of the order, namely Fischerellaceae (*Fischerella*, *Westelliopsis*), Mastigocladaceae (*Hapalosiphon*, *Westiella*, *Mastigocladus*) and Stigonemataceae (*Stigonema*), have been specifically studied with respect to bioactive metabolites. Arguably, this pursuit has been inherently hindered by inabilities to culture representative species of other taxa, as well as lack of clear links to either toxicity or drug potential which might, otherwise, fuel research in this regard. As one noteworthy example, in this regard, a novel species from the order was only recently isolated [10] as an epiphyte of the plant, *Hydrophilla verticillata*, and linked to intoxication of waterfowl (see Section 8, Relevance as Harmful Algae, below). However, the relevant toxic metabolites, in this case, remain to be identified.

## 3. Stigonematales as a Source of Bioactive Metabolites

Although metabolites typically classified as “cyanotoxins” are not generally linked to this family, there have notably been documented instances of members of the order producing known toxins. For example, a freshwater isolate of *Fischerella* was found [11] to produce the widely distributed hepatotoxin, microcystin-LR (MC-LR). Similarly, MC-LA was identified from a collection of a terrestrial species of *Hapalosiphon* [12]. And more recently, *Fischerella* isolated from a freshwater system in Australia was found [13] to produce several microcystin variants (*i.e.*, MC-LR, MC-LA, MC-LF, MC-FR and demethyl-MC-LR). In addition, the neurotoxic amino acid, β-methylamino-l-alanine (BMAA), was recently identified from an epiphytic stigonematalean species [14]. It was also previously found that a freshwater isolate of the species, *Umezakia natans*, apparently produced the hepatotoxic alkaloid, cylindrospermopsin; however, subsequent taxonomic analysis confirmed that this species belongs, in fact, to the Nostocales rather than the Stigonematales [15].

**Figure 2:**
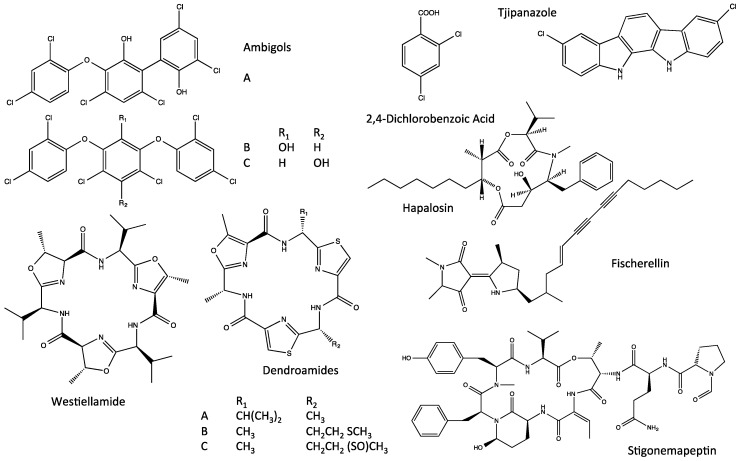
Examples of bioactive metabolites from the Stigonematales.

**Figure 3:**
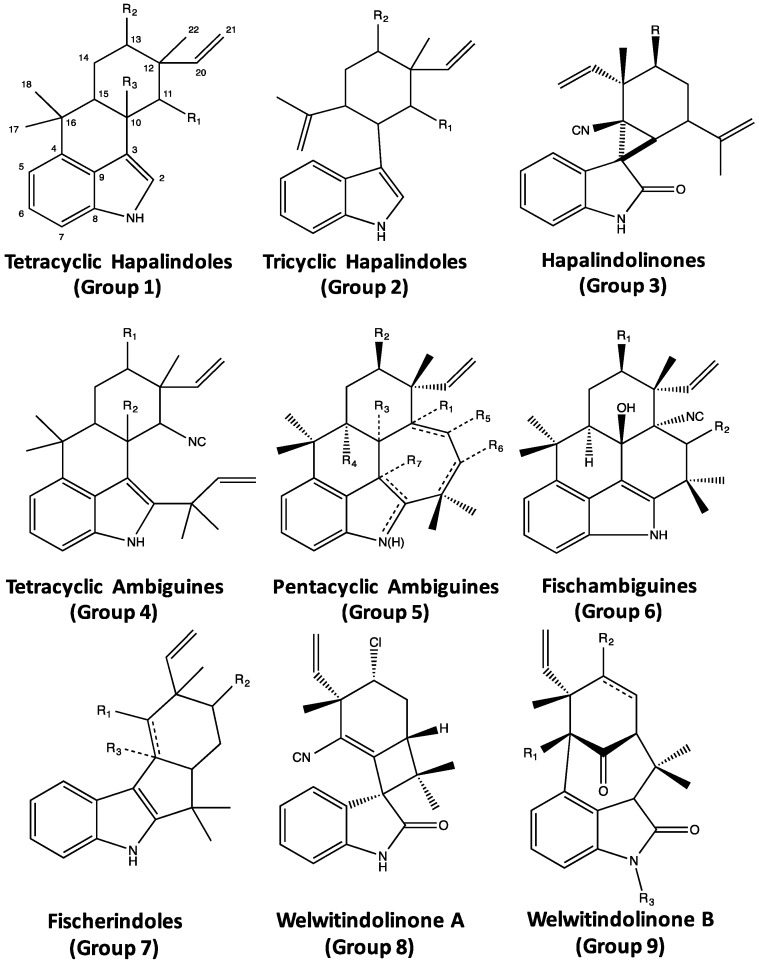
Classification scheme of indole alkaloids from Order Stigonematales. The previously described indole alkaloids are classified here as nine proposed groups; given are group number (as defined here), and corresponding chemical name of each group (based on previously named carbon skeletons). Note that stereochemistry is illustrated only for those sub-classes for which either a very limited number of variants have been identified (*i.e.*, Groups 3, 6 and 8), and/or no variability in stereodisposition (e.g., 12-epimers, C-10/11/15) has been yet reported (*i.e.*, Group 5 and 9). For more detailed explanation of variation within the subclasses, see Figure 4, Figure 5, Figure 6, Figure 7, Figure 8, Figure 9, Figure 10 and Figure 11.

That said, a wide range of bioactive compounds with both potential environmental (*i.e.*, ecological, environmental toxicology) and biomedical significance have been isolated and characterized from the Stigonematales (Figure 2). Interestingly, multiple polychlorinated aromatic compounds, specifically including the polyphenols ambigol A, B and C, 2,4-dichlorobenzoic acid and a carbazole alkaloid, tjipanazole D (Figure 2), have been isolated from the order; and, in turn, associated with diverse biological activity including antibacterial, anti-parasitic (*i.e.*, *Plasmodium, Trypanosoma*), molluscicidal and cytotoxic activity, as well as inhibition of cyclooxygenases (an enzyme responsible for inflammatory responses) and HIV-1 reverse transcriptase [16,17]. The diversity of chlorinated compounds, in particular, is perhaps notable in light of the very recent identification of a halogenase from the order (see Section 5, Biosynthesis of Indole Alkaloids from the Stigonematales) which is uniquely capable of chlorinating “freestanding” substrates [18]. More recently, studies of the capsular polysaccharides from the species, *Mastigocladus laminosus*, identified apparent cytotoxicity, as well as inhibition of cell migration and invasion, in a human carcinoma cell line, and specifically pointed to a role of secreted metalloproteinases in this regard; however, chemical characterization of the polysaccharides remains to be accomplished [19]. Not surprisingly, given the recognized diversity of non-ribosomal peptides from cyanobacteria, several bioactive peptides have been, likewise, identified from the Stigonematales, including several depsipeptides and bistratamides [20,21,22,23]. With respect to depsipeptides, hapalosin (Figure 2) was isolated from *Hapalosiphon welwitschii*, and specifically shown to inhibit P-glycoprotein-mediated multidrug resistance (MDR) activity more effectively, in fact, than clinically utilized verapamil [21]. And, more recently, stigonemapeptin (Figure 2), specifically containing the unusual amino acid, Ahp, *i.e.*, (3-amino-6-hydroxy-2-piperidone), was isolated from freshwater field samples of the genus, *Stigonema*, and found to act as a protease (*i.e.*, elastase, chymotrypsin) inhibitor [22]. “Bistratatamide-type” peptides (Figure 2) have also been isolated from order. Members of this unusual class of macrocyclic peptides containing thiazole and oxazole rings, and named for the bistratamides originally isolated from a marine worm *Lissoclinum bistratum* [24], have been isolated, in fact, from other cyanobacterial taxa (e.g., nostocyclamide from *Nostoc* spp. [25,26]). Representatives of the class from Stigonematales specifically include: (1) the dendroamides, isolated from *Stigonema dendroideum*, and shown—similar to hapalosin—to reverse MDR via P-glycoprotein inhibition [23]; and (2) westiellamide, isolated from *Westiellopsis prolifica*, and shown to be cytotoxic toward human epithelial (KB) and colon (LoVo) cancer cell lines [20]. Finally, a considerable diversity of alkaloids has been isolated from the Stigonematales. Among these, the enediyne-containing fischerellins (Figure 2) were isolated from *Fischerella muscicola*, and characterized by a wide range of bioactivities including antialgal, herbicidal and antifungal activity, as well as toxicity towards rotifers and crustaceans [27,28]. Of the alkaloids isolated from the order, however, a family of indole alkaloids (Figure 3) is clearly the most chemically diverse, important and widespread group of compounds [29,30,31,32,33,34,35,36,37,38,39,40,41,42,43,44,45], and is the primary focus of the remainder of this review.

## 4. Indole Alkaloids from the Stigonematales: Chemical Diversity

A chemically diverse family of indole alkaloids (Figure 3) produced by the Stigonematalean cyanobacteria, with more than 80 variants reported so far [29,30,31,32,33,34,35,36,37,38,39,40,41,42,43,44,45], arguably represent the most characteristic bioactive secondary metabolites from the order. And, in fact, these metabolites have not been found outside of the Stigonematales. Although diverse, these molecules all bear certain structural similarities, likely owing to shared biosynthetic origins (see Section 5, Biosynthesis of Indole Alkaloids from the Stigonematales) including: a polycyclic, *i.e.*, tricyclic, tetracyclic or pentacyclic, ring-system, specifically based on an indole core; a pendant vinylic group; and, in almost all cases, a potentially reactive functional group comprised of either isonitrile or isothiocyanate—or, in a relatively few cases, nitrile and thiocarbamate. In addition, site-specific chlorination is frequently observed among the variants. Based on carbon skeletons observed so far, indole alkaloids from the Stigonematales can be effectively classified into nine structural groups (Figure 3). Thus far, however, these alkaloids have been only identified from four genera of the Stigonematales (*i.e.*, *Fischerella*, *Hapalosiphon*, *Westiella*, *Westiellopsis*), but as they span multiple families within the order, it is likely that related metabolites remain to be identified from others.

### 4.1. Hapalindoles

Of the indole alkaloids produced by the Stigonematales, the hapalindoles are the largest and most widespread group, and ostensibly represent the archetypal sub-class of the indole alkaloids from the order [29,30]. In fact, biosynthesis studies suggest that hapalindoles are the precursors to other sub-classes of the so-called *hapalindole-type alkaloids* from the Stigonematales. First identified more than 30 years ago by Moore *et al.* [29] from *H. intricatus*, specifically based on a suggested allelopathic role (see Section 7, Possible Ecological Function), a diversity of hapalindoles have been since isolated from numerous species of *Hapalosiphon,* as well as *Fischerella* and *Westelliopsis*.

**Figure 4:**
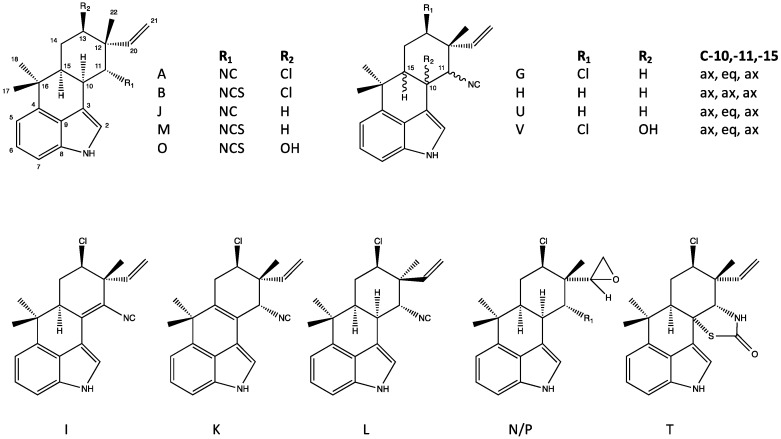
Previously described tetracyclic hapalindoles (Group 1) from Stigonematales.

**Figure 5:**
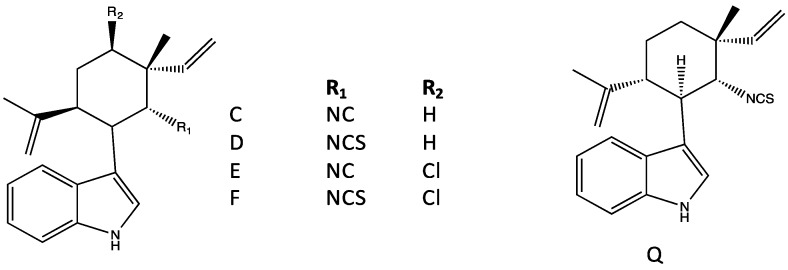
Previously described tricyclic hapalindoles (Group 2) from Stigonematales.

Structurally, the hapalindoles are further sub-classified into tetracyclic (Group 1) and tricyclic (Group 2) variants—with the latter being more common—and, within both of these sub-classes, there is considerable variation including both chlorinated and non-chlorinated (at C-13; see Figure 4 and Figure 5), and occasionally hydroxylated (e.g., hapalindole O), representatives. In some rare cases (e.g., hapalindole N and P), the vinylic group is replaced by an epoxide. Moreover, all members of the sub-class and, more generally, nearly all members of the larger class identified so far, contain reactive functional groups at the C-11 (Figure 4 and Figure 5) position; in this regard, isonitrile- and isothiocyanate-bearing hapalindoles are, by far, the most common, but thiocarbamates (e.g., hapalindole T) [31] have also been identified. Finally, within the group, there is variability with respect to stereochemistry most notably including epimers at the C-12 position, and the diastereomers at the C-10 and C-15 positions (Figure 4). Chemical variants of the hapalindoles (and, indeed, other sub-classes) are generally named alphabetically (*i.e.*, hapalindole A, B, C, *etc.*) in order of their identification with most of the core structures (hapalindoles A, B, C–Q and T–V) notably identified by Moore *et al.* [29,30]; however, additional nomenclature (e.g., 12-*epi*-hapalindoles, deschloro hapalindoles) are also frequently found in the literature to further elaborate structural variants. As an example, hapalindole A is a tetracyclic, chlorinated isonitrile-containing congener, whereas hapalindole B is the corresponding isothiocyanate. Hapalindole J and M, in turn, are the non-chlorinated isonitrile and isothiocyanate congeners, respectively (Figure 4), whereas 12-*epi*-hapalindole J has been identified [32] as an epimer of the former.

**Figure 6:**
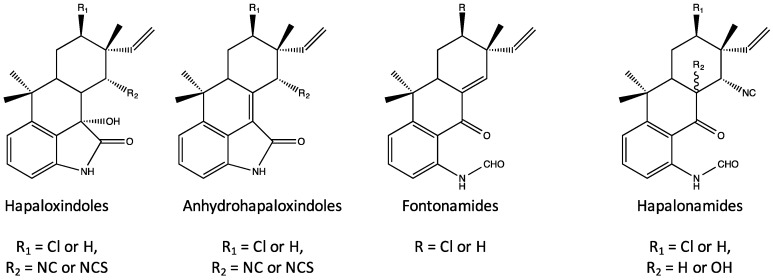
Oxidized hapalindoles isolated from Stigonematales.

Notably alongside hapalindoles from *H. fontinalis*, Moore *et al.* [30,33] isolated several more polar, minor constituents, namely fontonamides, hapalonamides and hapaloxindoles (Figure 6). It was experimentally shown [30] that these congeners were likely oxidation products of hapalindoles, and specifically hapalindole A, formed by singlet oxygen with hapaloxindole serving as a precursor for fontonamide and hapalonamide, whereby subsequent oxidation leads to opening of the indole-ring in the latter (Figure 6). These oxidation products are seemingly widespread as they have also recently been found [34], alongside hapalindoles, from *Fischerella*. Moreover, an oxindole ring is similarly found in other sub-classes including hapalindolinones and welwitindolinones (Figure 3) suggesting a similar role of oxidation in the observed chemical diversity of these groups.

### 4.2. Hapalindolinones

The hapalindolinones (Group 3) represent a unique structural sub-class containing a spiro-fused cyclopropane ring, and oxindole ring. Hapalindolinones A and B (chlorinated and non-chlorinated, respectively; see Figure 3) were, in fact, originally identified from a species of *Fischerella* isolated from a Florida Everglades soil sample [35]. It is tempting to speculate that hapalindolinones may represent derivatives of the tricyclic hapalindoles, specifically formed via cyclization of C-11 to yield the cyclopropane ring, and singlet oxygen oxidation (as suggested for hapaloxindole derived from tetracyclic hapalindole A, see above) to form the oxindole ring. However, the biosynthesis of this subclass has yet to be investigated, and the hapalindoles are classified separately here, in part, because of their unique biological activity (Section 6, Biological Activity of the Hapalindole-Type Alkaloids).

### 4.3. Ambiguines

**Figure 7:**
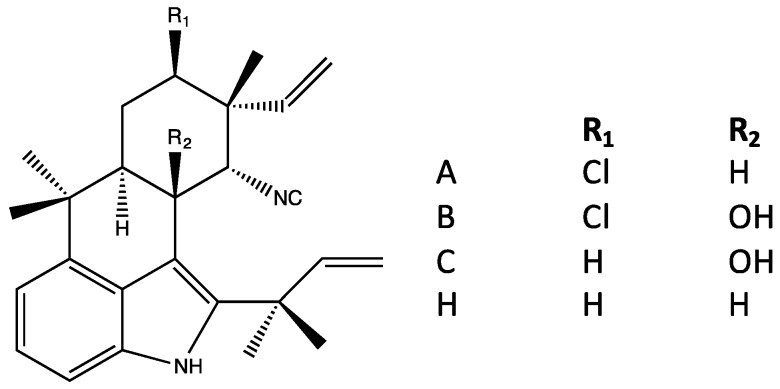
Previously described tetracyclic ambiguines (Group 4) from Stigonematales.

**Figure 8:**
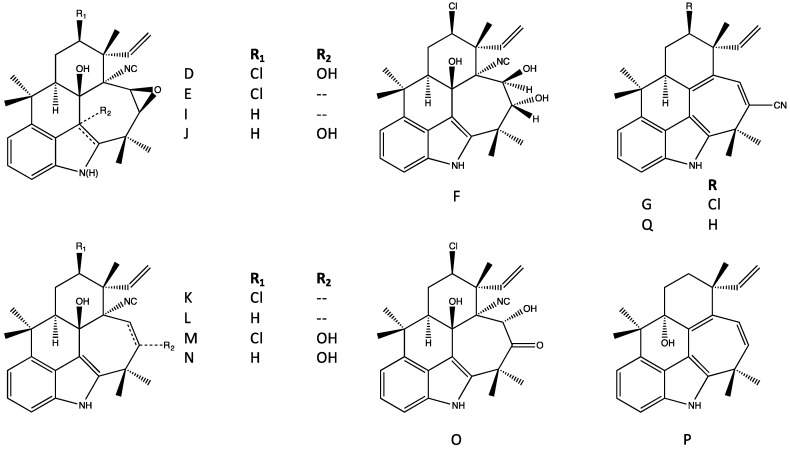
Previously described pentacyclic ambiguines (Group 5) from Stigonematales.

The second largest group of indole alkaloids of Stigonematales is the ambiguines. Members of this class are specifically distinguished by an isoprene unit attached at the C-2 position of the indole ring [36,37,38] including tetracyclic and pentacyclic variants with the latter formed by cyclization of the isoprene via an epoxide bridge (Figure 7 and Figure 8, respectively), although hydroxyl, carbonyl and even nitrile groups may replace the epoxide. Also similar to the hapalindoles, both chlorinated and non-chlorinated congeners are common. In addition, hydroxyl-containing variants—specifically at the C-10 (Figure 8)—of the ambiguines are quite common. Unlike hapalindoles, no isothiocyanate-containing variants are known, however, interestingly nitrile-bearing congeners of both tetracyclic [39] and pentacyclic (*i.e.*, ambiguine G [40]) ambiguines have been identified. Moreover, like hapalindoles, 12-epimers exist. Specifically, Walton *et al.* [39] recently identified a 12-*epi*-ambiguine B nitrile from an isolate of *Fischerella*, and subsequent studies have, likewise, identified the related, non-chlorinated congeners (e.g., 12-*epi*-ambiguine C nitrile [41]) from the same strain.

The ambiguines were first isolated by Smitka *et al.* [36] who identified six variants, ambiguines A–F isonitriles, including both tetra- and pentacyclic structures, from three separate genera and species (A–F from *F. ambigua,* A and E from *H. hibernicus* and D and E from *W. prolifica*). Subsequently, Huber *et al.* [40] isolated ambiguine G (from *H. delicatulus*), and specifically identified it as a novel nitrile-bearing variant. Identification of additional variants, *i.e.*, ambiguines H–Q, followed [37,38,42]. Notable among these, ambiguine P isolated from *F. ambigua* represents an unusual variants lacking the typical isonitrile, or less common nitrile, found among ambiguines (or alternative functional group, e.g., isothiocyanate, thiocarbamate, present in other indole alkaloids from the order).

### 4.4. Fischambiguines

**Figure 9:**
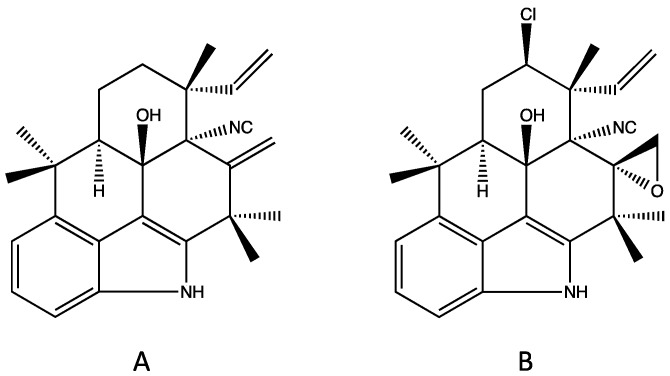
Previously described fischambiguines (Group 6) from Stigonematales.

Clearly related to, but classified separately here from, the ambiguines are fishambiguines. This pentacyclic sub-class is distinguished by the presence of a six-membered (rather than 7-membered) cyclization of the C-2 isoprene group (Figure 9). Fischambiguines A and B were first isolated, rather recently, from *F. ambigua* by Mo *et al.* [42], specifically based on weak antibacterial activity. And, to date, only two variants (A and B) have been characterized, and specifically differ based on the presence of either an exomethylene (=CH_2_) carbon or epoxide, respectively (Figure 9). Similar to other sub-classes, the fischambiguines include both chlorinated (e.g., fischambiguine B) and non-chlorinated (e.g., fischambiguine A) congeners.

### 4.5. Fischerindoles

**Figure 10:**
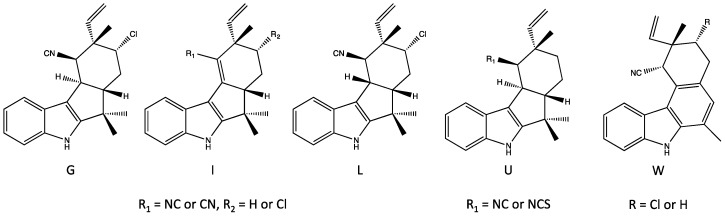
Previously described fischerindoles from Stigonematales.

Fischerindoles, first isolated from *F. muscicola* by Park *et al.* [43], are tetracyclic alkaloids considered to be chemically related to the hapalindoles, but specifically distinguished by ring-fusion at the C-2 (rather than C-6) of the indole ring forming a typically five-membered C-ring (Figure 10), although exceptions to this have been reported (see below). Similar, to the hapalindoles, the fischerindoles include both isonitrile- or isothiocyanate-containing (at C-11, see Figure 10), as well as chlorinated and non-chlorinated congeners (at C-13, see Figure 10), and all of the same stereocenters (*i.e.*, C-10/11 and 12-epimers) found among the hapalindoles are, likewise, found in the fischerindoles. Most interestingly perhaps, this includes 12-epimers as the apparently most common stereoisomer. As an example, fischerindole L, the first representative isolated, was named (similar to all subsequent variants) on the basis of similarity to the stereochemistry of hapalindole L (a 12-epimeric variant) that was isolated in the same study [43]. And notably, in fact, hapalindole L and fischerindole L have similar antifungal properties (see Section 6, Biological Activity of the Hapalindole-Type Alkaloids). Interestingly, in addition to the typical isonitrile and isothiocyanate variants, nitrile-bearing fischerindoles have been identified [44]. Moreover, variants (*i.e.*, fischerindole W) characterized by a six-membered (rather than 5-membered) aromatic C-ring have been recently described [44]; however, unlike other sub-classes and variants, no biological activity has been reported, and little is, otherwise, known about these unusual congeners.

### 4.6. Welwitindolinones

**Figure 11:**
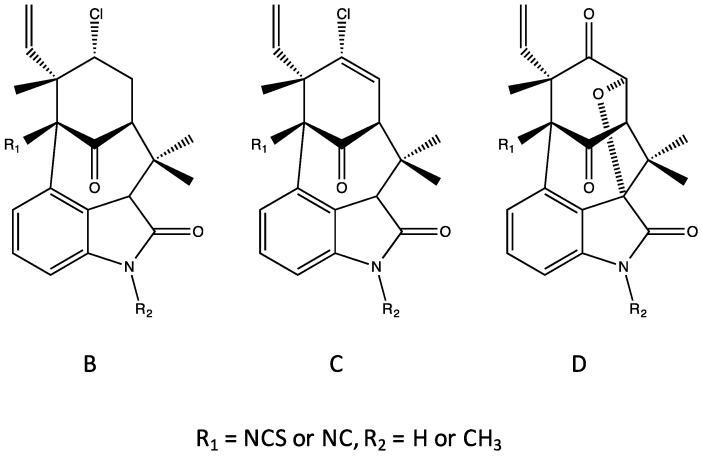
Previously described Type B welwitindolinones (Group 9) from Stigonematales.

Finally, one of the most chemically unique sub-classes are the tetracyclic indolinone-containing welwitindolinones (Figure 3 and Figure 11). First isolated from *Westiella intricata* and *H. welwitschii* by Stratmann *et al.* [45], the group is further sub-categorized into Type A and B subclasses with the latter being the most common. In fact, the former currently includes only the unusual cyclobutane-containing welwitindolinone A. The remaining welwitindolinones (B–D) are classified as Type B (Figure 11) that represent considerable structural variability; however, it has been suggested that all welwitindolinones are biosynthesized from the cyclobutane-containing welwitindolinone A as a common precursor. Interestingly, all of the welwitindolinones, with the exception of the unusual and highly oxidized welwitindolinone D (see below), are chlorinated, and similar to other sub-classes, all known variants have either an isonitrile or an isothiocyanate (at C-11; see Figure 11). More notably, multiple variants, specifically of welwitindolinone C, are characterized by N-methylation of the indole ring (Figure 11). Owing to the remarkable scaffold of the welwitindolinones, as well as their emerging potential as anticancer drugs (see Section 6, Biological Activity of the Hapalindole-Type Alkaloids), considerable effort has been placed on the enantiospecific total synthesis of these compounds [46,47].

Similar to the hapalindoles, several oxidized products of welwitindolinones have been isolated [48]. These include the hydroxylated 3-hydroxy-*N*-methylwelwitindolinone C isothiocyanate and isonitrile, and the unusual, non-chlorinated *N*-methylwelwitindolinone D isonitrile. It was specifically proposed that these variants were derived from photooxidation of the previously isolated *N*-methylwelwitindoline C isonitrile [48].

## 5. Biosynthesis of Indole Alkaloids from the Stigonematales

In support of the impressive chemical diversity observed, a continuing body of knowledge has evolved with respect to the biosynthesis of the hapalindole-type alkaloids. Species (and strains within species) of the Stigonematales frequently produce several of the indole alkaloid sub-classes, and the observed isolation of diverse congeners from a single source leads to the conclusion that a similar biosynthetic pathway occurs for these compounds. Indeed, a unified biosynthesis of the hapalindole-type alkaloids was accordingly proposed soon after their discovery [45]. However, very recent genomic data have led, in many cases, to considerable revision of this proposed biogenetic pathway.

**Figure 12:**
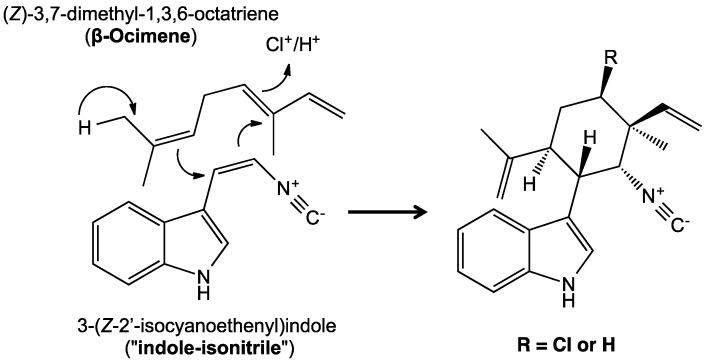
Originally proposed biosynthesis of a tricyclic hapalindole, as an intermediate for other hapalindole-type alkaloids (see Figure 14), via chloronium or H^+^ induced condensation [45]. Recent molecular studies, however, suggest an alternative biosynthetic route [55].

In the first assessments of the biosynthesis of the hapalindole alkaloids, Stratmann *et al.* [45] specifically suggested that initial steps occurred (Figure 12) by way of a chloronium or hydrogen ion-induced condensation of 3-(*Z*-2′-isocyanoethenyl)indole (*i.e.*, “indole-isonitrile”) and (*Z*)-3,7-dimethyl-1,3,6-octatriene (*i.e.*, β-ocimene), whereby these precursors are formed, respectively, from tryptophan and geranyl pyrophosphate (GPP), leading to the formation of a presumptive tricyclic hapalindole. Based on this biosynthetic scheme, 12-epimers of the hapalindoles were proposed to be alternatively derived from either *E-* or *Z-*isomers of β-ocimene (see [49]). Very recent studies, incorporating genomic and other molecular approaches, however, clearly suggest an alternative biosynthetic route, and specifically direct prenylation of the indole-isonitrile by GPP, rather than involvement of β-ocimene (as discussed below). Similarly, the presence of a *cis* and *trans* isomers (*i.e.*, *Z*- and *E-*isomers, respectively) of the indole-isonitrile were proposed to underlie the stereochemistry at C-10/11 in hapalindoles; however, very recent genomic studies suggest that only the *Z*-isomer is a substrate for the condensation step (also discussed below).

**Figure 13:**
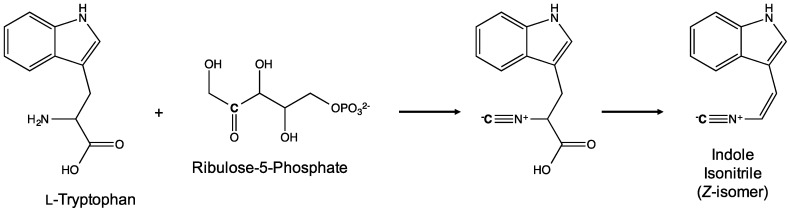
Biosynthesis of the indole-isonitrile precursor as inferred based on the identification of the WelI1-3 and AmbI1-3 genes as homologs of previously characterized isonitrile synthase genes, and subsequent *in vitro* studies of gene products [49,54].

With respect to the biosynthesis of the indole-isonitrile, it was proposed in early studies that the isonitrile functional group found in this precursor, and consequently, the majority of the sub-classes, is either derived from inorganic cyanide, or cyanide produced from C-2/N-2 of amino acids [50]—as reported, in fact, for cyanobacteria [51]—by way of the tetrahydrofolate (THF) C1 pathway, and specifically a 5-formimino-THF intermediate. Contribution of amino acid-derived C and N to the isonitrile is supported by studies of hapalindole A, and specifically feeding studies of *H. fontinalis* utilizing isotopically (*i.e.*, ^13^C, ^15^N) labeled glycine [52]. The incorporation of inorganic cyanide is indirectly supported by feeding studies of sponge-derived, isonitrile-containing compounds [53], as well as radiolabeling (*i.e.*, ^14^C-labeled cyanide) studies in the cyanobacteria. However, attempts to replicate the latter feeding studies with inorganic cyanide labeled with stable isotope (*i.e.*, ^13^C) were confounded, as the requisite concentrations proved toxic to the cyanobacterial cultures [52]. Moreover, recent genomic studies [54] suggest an alternative biogenic route for the indole-isonitrile. Identification of a gene cluster (*amb*) associated with ambiguine biosynthesis revealed three homologs (*i.e.*, AmbI1-3) of previously characterized, bacterial isonitrile synthase genes, and following purification of the gene products—and *in vitro* enzyme studies—demonstrated specific production of the *cis*-isomer (and, notably, not the *trans* form) of the indole-isonitrile from tryptophan and ribulose-5-phosphate (as a carbon source) as shown in Figure 13 [54]. Similar results were found for homologs from the *wel* gene cluster associated with biosynthesis of welwitindolinones [49]. These studies, therefore, support an alternative route which does not utilized cyanide. Specifically, in this proposed pathway, the nitrogen of the isonitrile is derived, instead, from tryptophan, and the carbon from a donor (*i.e.*, ribulose-5-phosphate in feeding studies); and subsequent oxidative decarboxylation, in turn, yields the indole-isonitrile intermediate (Figure 13).

The origin of the isothiocyanate found in many of the variants, on the other hand, has yet to be investigated. Several feeding studies of isonitrile- and isothiocyanate-containing terpenoids produced by marine sponges generally suggest inter-conversion between cyanide and thiocyanate, and subsequent incorporation to produce the corresponding isonitriles and isothiocyanates, respectively [53]. However, the aforementioned genomic studies [54] which argue against incorporation of cyanide, and rather donation of carbon to the amine of tryptophan, would, in general, conflict with this proposal. An alternative hypothesis proposes the subsequent incorporation of sulfur into the isonitrile [49,53]; and, most recently, it was suggested, based on a the lack of a dedicated gene within biosynthetic gene clusters, that either a “pathway independent sulfur carrier protein,” or alternative enzymes, such as a cysteine desulfurase, could be responsible for isothiocyanates found in several hapalindole-type alkaloids [49]. Likewise, as to the rare nitrile variants, specifically as found in ambiguines and fischerindoles, no studies have investigated the biosynthetic origin of this group, and previous studies simply propose an otherwise unexplained “rearrangement” of the isonitrile [40].

According to the originally proposed scheme (Figure 12), therefore, the first step in the common biosynthetic pathway might yield one of four of the tricyclic hapalindoles (*i.e.*, C–F), depending on whether an isonitrile or isothiocyanate indole precursor, was involved (*i.e.*, C/E or D/F, respectively), and whether a hydrogen or chloronium ion was utilized in the condensation (*i.e.*, C/D or E/F, respectively). That said, recent molecular studies cast doubt on the role of chloronium ion-induced condensation, and suggest instead an enzymatically driven condensation reaction followed by subsequent enzymatic halogenation of the resulting hapalindole [18,55]. In this case, either hapalindole C or D (*i.e.*, isonitrile and isothiocyante variants, respectively) would be the expected tricyclic precursor, but if subsequent introduction of sulfur to the isonitrile (to produce the isothiocyanate) is assumed, and which is supported by recent data, then hapalindole C is the most likely tricyclic intermediate. However, if stereochemistry of the indole-isonitrile/isothiocyanate or monoterpenoid precursors does explain the existence of various stereoisomers (*i.e.*, 12-epimers, C10/11/15 isomers), as has been propose, it is likely that other, yet to be identified, intermediates might exist.

**Figure 14:**
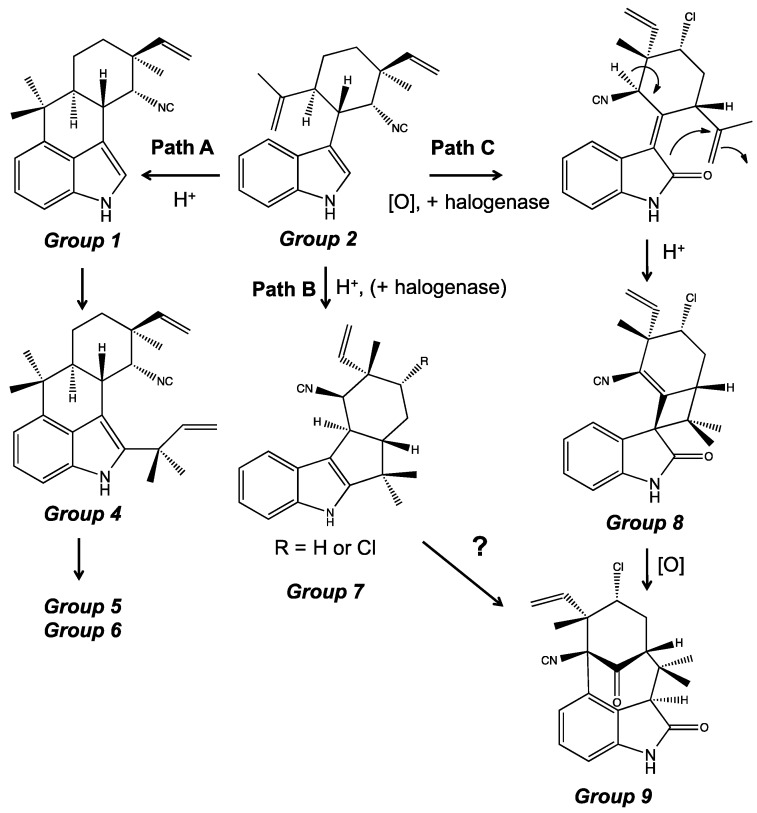
Biosynthesis of indole alkaloid sub-classes from a tricyclic precursor. The scheme shown is specifically based on a hapalindole C (*i.e.*, isonitrile-bearing) precursor.

After the formation of a tricyclic intermediate, and thus Group 2 alkaloids, the proposed biosynthetic scheme diverges into three paths as shown in Figure 14. The first path suggests the cyclization between the C-4 carbon in the indole ring, and the C-16 carbon in the isopropylidene group [45,56]. The cyclization would, thereby, produce the tetracyclic hapalindoles of Group 1. The formation of oxindoles and formamides (Figure 6) are proposed to occur through oxidation of these tetracyclic hapalindoles. Notably, no studies have, to date, elucidated the biosynthetic origin of the hapalindolinones (Group 3), although it is assumed to occur via oxidation (to form the oxindole ring), and subsequent rearrangement (to form the spiro-fused cyclopropane ring). Prenylation of C-2 of the indole ring (of a tetracyclic hapalindole precursor) would produce tetracyclic ambiguines from Group 4. And if the isoprene group reacts further, intermolecular cyclization would lead to the pentacyclic ambiguines and fischambiguines of Group 5 and 6. Oxidation, and various rearrangement of these compounds, would subsequently afford variants within the ambiguines sub-classes.

The second pathway would support cyclization of the tricyclic hapalindole between C-2 of the indole ring, and C-16 of the isopropylidene group, producing the tetracyclic fischerindoles of Group 7. The condensation of the hapalindole would presumably occur via acid catalysis, and be possibly governed by an enzymatic reaction. Park *et al.* [43] showed that the cyclization reaction was possible without the presence of an enzyme using hapalindole C formamide as a starting product. A strong acid was used to convert hapalindole C formamide to fischerindole C formamide and fischerindole C amine (Bonjouklian *et al.*, 1988). Using the same conditions, however, this same reaction was not reproducible with hapalindole E formamide that only differs from hapalindole C formamide by presence of a chlorine atom. Recent identification of a halogenase [18] capable of utilizing fischerindole as a substrate, however, suggests chlorination could occur post-cyclization. Moreover, the identification of novel fischerindoles (Figure 10) with 6-membered “B-rings” suggest an alternative, but yet investigated, biosynthetic route.

Finally, a third pathway suggests the oxidation of the tricyclic hapalindole to produce an intermediate indolinone with a structure essentially similar to a tricyclic variant of anhydrohapaloxindole A. Anhydrohapaloxindole A has been shown to be produced from singlet oxygen oxidation of hapalindole A [30,33], and similar oxidation of a tricyclic hapalindole could presumably occur. According to the biosynthesis proposed by Stratmann *et al.* [45] this intermediate would then undergo intermolecular cyclization between C-3 of the indole ring, and C-16 of the isopropylidene group, via acid catalysis to produce welwitindolinone A from Group 8. In order to form the remaining welwitindolinones from Group 9, it was proposed that an isocyano epoxide intermediate would be formed, and further intermolecular rearrangements would occur. Subsequent, oxidation and methylation of the welwitindolinones would, in particular, produce variants such as the oxidized welwitindolinones including 3-hydroxy-*N*-methylwelwitindolinone C isonitrile. That said, it is worth noting that more recent assessments have proposed an alternative pathway for the welwitindolinones, and specifically oxidative ring contraction of 12-*epi*-fischerindole I to give welwitindolinone A [56], however, this has yet to be investigated.

As previously mentioned, only very recently have relevant gene clusters linked to the biosynthesis of the indole alkaloids been elucidated [49,54,55,57,58]. Work by Hillwig *et al.* [49,54] identified gene clusters from *F. ambigua* and *H. welwitschii*, and established a link to the biosynthesis of ambiguine and welwitindolinone alkaloids, respectively. The 42 kbp *amb* gene cluster, encoding 32 proteins, was confirmed by both bioinformatics, and *in vitro* enzyme studies, to be involved in the biosynthesis of ambiguines including steps common to all hapalindole alkaloids, and specifically genes associated with biosynthesis of tryptophan, prenylation precursors (*i.e.*, isopentyl pyrophosphate and dimethylallyl pyrophosphate [DMAPP]) and the indole-isonitrile intermediate, as well as key steps in the prenylation of tetracyclic hapalindoles to ambiguines [54]. Furthermore, the presence of several oxygenases suggests a contribution to the “late stage” biosynthetic steps characteristic of the diversity of ambiguines. Concurrently, characterization of the *wel* cluster from *H. welwitschii* [49] identified genes related to the biosynthesis of the welwitindolinones including key precursors, namely of 3-(*Z*-2′-isocyanoethenyl)indole, and the putative monoterpenoid building blocks. Gene products associated with indole-isonitrile biosynthesis (*i.e.*, AmbI1-3 and WelI1-3) in both *amb* and *wel* gene clusters were evaluate in enzyme studies, and specifically found to produce only the *cis* form of the indole-isonitrile intermediate (Figure 13). Finally in addition to these common genes, the *wel* gene cluster included apparent methyltransferases suggested to have a role in the *N*-methylation of several of the welwitindolinones, and were demonstrated in *in vitro* enzyme studies to, in fact, function as such. Most generally, both studies support condensation of geranyl pyrophosphate (GPP) and the indole-isonitrile, rather than conversion of GPP to β-ocimene, as previously proposed [45,56].

**Figure 15:**
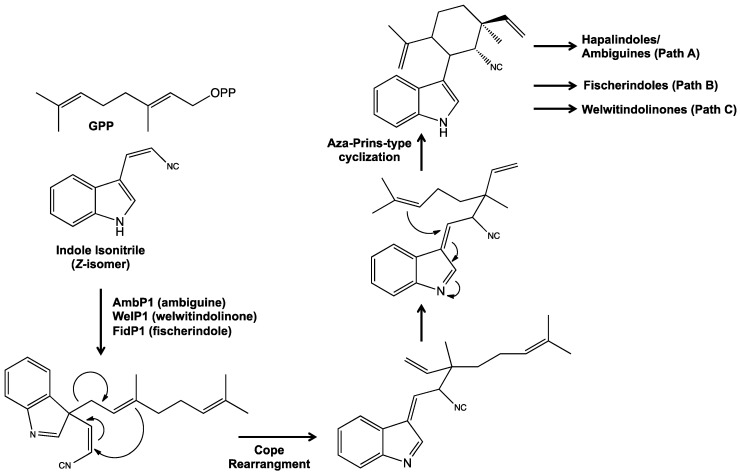
Most recently proposed pathway for synthesis of common tricyclic hapalindole precursor, via a monogeranylated indolenine intermediate, as supported by recent molecular studies [55]. Subsequent steps to yield other alkaloids would occur via Paths A, B or C as shown in Figure 14.

A particularly comprehensive, subsequent study [57] coupled whole genome sequencing of multiple stigonematalean species—including *Fischerella*, *Hapalosiphon* and *Westiella*—and previously published, and unpublished, sequence data for gene clusters associated with hapalindole-type alkaloid biosynthesis (e.g., *amb*, *wel* [49,54]) to elaborate a unified biosynthetic pathway. Comparative studies, accordingly, identified 19 gene sets, with 92% sequence identity at the protein level, which were common to all gene clusters (and species/strains) investigated. Supporting the proposed early steps of the biosynthesis of the alkaloids, these “core” genes include those involved in tryptophan biosynthesis, and isonitrile biosynthesis genes involved in the formation of the 3-(*Z*-2′-isocyanoethenyl)indole precursor. Notably in this study, the gene products of the latter, and specifically WelI1/3, were found to produce *in vitro* both *cis* and *trans* isomers of the indole-isonitrile, and were thereby suggested to explain the stereodisposition between C10 and C11 observed among the hapalindole-type alkaloids. However, concurrent studies suggested (as discussed above) that only the *cis* form (*i.e.*, *Z*-isomer) is produced, and subsequently utilized, in the condensation [49,55]. This discrepancy was attributed to differences in incubation time of feeding experiments in the two studies. However, it should be noted that WelI genes assessed in the conflicting studies [49,57] were, in fact, isolated from two different genera/species (*i.e.*, *H. welwitschii* and *W. intricate*), and differ to some extent in their sequence; and it is possible, therefore, that differences in enzymatic activity could also reflect phylogenetic differences in the biosynthesis. Conserved genes additionally included those involved in isoprene biosynthesis, specifically including geranyl phosphate synthases, as well as aromatic prenyltransferases which was proposed to be involved in the condensation of the indole-isonitrile to GPP (Figure 15) although this could not be confirmed in the study. In addition to these common genes, several others seemingly specific to the different sub-types were identified including various putative oxygenases, an aromatic prenylase involved in the C-2 prenylation of the ambiguines (from the *amb* cluster), and methyltransferases involved in the *N*-methylation of the welwitindolinones (from the *wel* cluster). Finally, a phylogenetic analysis of the strains and the gene clusters—based on both 16s rDNA and relevant, conserved biosynthetic genes (*i.e.*, prenyltransferase) —indicated a monophyletic group, and thus vertical inheritance, of these biosynthetic genes. This latter finding, thereby, further supports the unique distribution of the indole alkaloids within the Stigonematales.

Two very recent studies, based on genomic data, and *in vitro* enzymatic studies, have attempted to elucidate a potential mechanism for condensation of GPP and the indole-isonitrile precursor [55,58]. In one study, a homologous series of strictly magnesium-dependent aromatic prenyltransferases (AmbPI, WelP1 and FidP1) was identified across related biosynthetic gene clusters (*i.e.*, *wel*, *amb* and unpublished *fid*), and specifically shown to direct the formation of 3-geranyl 3-isocyanovinyl indolenine—via condensation of GPP and the *Z*-isomer of the indole isonitrile—as a common intermediate [55]. Following an unusual Cope rearrangement, it was suggested that a tricyclic hapalindole could be formed via aza-Prins-type cyclization, and subsequently provide a starting point for a wide range of hapalindole-type alkaloids (Figure 15). Concurrent studies, however, by Li *et al.* [58] identified the *fam* gene cluster from *F. ambigua* (involved in the biosynthesis of ambiguines), and demonstrated through *in vitro* studies with cell-free lysates, the role of two prenyltransferases (FamD1 and FamD2) and a cyclase (FamC1) within this cluster. It was proposed that FamD2 and FamC1 “act in concert” to catalyze the condensation of the indole-isonitrile and GPP via similar Cope rearrangement, and a similar quaternary intermediate, but distinct from the studies by Liu *et al.* [55] suggested the formation of a tetracyclic hapalindole core, and namely 12-*epi*-hapalindole U. Subsequent to this, FamD1 was shown to prenylate (with DMAPP) at the C-2 position to give rise to the ambiguines. Furthermore, it was suggested that the cyclase step (via FamC1, or perhaps homologs) could be involved in determining stereochemistry (*i.e.*, C-12 epimers, C10/C15), and also directing biosynthesis toward the fischerindoles. These findings, in general, reinforce a biosynthesis that circumvents the previously proposed β-ocimene precursor in this key step. Intriguingly, both studies point to dependence of the condensation step on pH and/or Mg^2+^, and might, thereby, underscore potential plasticity, relevant to structural diversification, at this step.

Finally, recent work from the Liu group [18] has identified a non-heme, iron-dependent halogenase (*i.e.*, *welO5*) from the *wel* gene cluster, and has specifically demonstrated the ability of this enzyme to catalyze chlorination of the C-13 of a tricyclic hapalindole (*i.e.*, 12-*epi*-hapalindole C) and fischerindole (*i.e.*, 12-*epi*-fischerindole U). This finding not only explains the presence of chlorinated and non-chlorinated congeners found among many of the hapalindole-type alkaloids, but also suggests a role of this chlorination as a first committed step in the subsequent formation of welwitindolinones [18] (Figure 14), and further explains, therefore, the previously unexplained absence of dechloro variants within this sub-class.

## 6. Biological Activity of the Hapalindole-Type Alkaloids

The indole alkaloids from the Stigonematales have been generally associated with a wide range of biological activity (Table 1) which is the focus of the remainder of this review. The reported biological activity suggests a role of these compounds in the chemical ecology of the order, as well as both environmental and biomedical relevance as potential toxins and promising leads to drug discovery, respectively. As to the potential as drugs, a preponderance of synthesis studies has, accordingly, focused on these alkaloids, and is reviewed extensively elsewhere [47].

### 6.1. Antimicrobial Activity

Arguably, the primary bioactivity of the hapalindole-type has been inhibition of microbes including bacteria, fungi and algae. In fact, the hapalindoles, as the archetypal member of the class, were first isolated from *H. fontanalis* based on antimicrobial—and specifically antialgal—activity of extracts [29,30]. Subsequent studies, in the course of identifying new variants, have confirmed antimicrobial activity of both tri- and tetracyclic hapalindoles [34,42,69]. Most recently, Kim *et al.* [34] reported the isolation of several hapalindoles including a novel variant, hapalindole X, and demonstrated relatively potent activity (MIC ≥ 2.5 μM) of this congener against *Mycobacterium tuberculosis*, and the yeast, *Candida albicans*, as well as potent activity of several other hapalindoles (*i.e.*, A, I, J) and presumptive oxidative products (*i.e.*, hapalonamide H). It is noteworthy that both chlorinated and non-chlorinated congeners (e.g., hapalindole A and J) display similar antimicrobial activity [34]. Furthermore, comparable antimicrobial activity has been reported for 12-*epi*-hapalindoles [62,64]. Relatively limited information exists, on the other hand, as to the role of the variable C-11 functional group of the hapalindoles; in particular, studies have generally focused on the more common isonitrile-bearing variants compared, for example, to the less common isothiocyanate congeners. Moore *et al.* [29], in the original isolation of the hapalindoles, did indicate that hapalindole A “was responsible for most of the antialgal and antimycotic activity of *H. fontinalis*,” and reported no biological activity for hapalindole B, the isothiocyanate equivalent, isolated as a minor component in the same study; however, no specific bioactivity data were, in fact, presented in this study. That said, the thiocarbamate-containing variant, hapalindole T, was found to be antibacterial against a range of Gram-positive and -negative representatives, and quantitatively comparable to other hapalindoles in this regard [31].

In addition to inhibition of heterotrophic bacteria and fungi, it has been shown that hapalindoles effectively inhibit the growth of other algae, and cyanobacteria, in particular. Etchagaray *et al.* [63] specifically used antialgal activity to guide isolation of several metabolites from an Amazonian isolate of *Fischerella,* and identified two putative hapalindoles, including the 12-*epi*-hapalindole F (as confirmed by NMR studies), which inhibited a representative strain of the cyanobacterial genus, *Synechococcus*. This finding is notable in that hapalindole F is an isothiocyanate-bearing variant; however, Doan *et al.* [62] also demonstrated inhibition of cyanobacteria and eukaryotic green algae by the isonitrile counterpart of 12-*epi*-hapalindole F (*i.e.*, 12-*epi*-hapalindole E).

Similar to the hapalindoles, Smitka *et al.* [36] originally isolated ambiguines based on inhibition of several representative fungi including *Aspergillus oryzae, Candida albicans, Penicillium notatum, Saccharomyces cerevisiae* and *Trichophyton mentagrophytes*. And subsequent studies [37,38,42] have, likewise, demonstrated diverse antibacterial and antifungal activity of both tetra- and pentacyclic ambiguines encompassing rather diverse structural variability within the sub-class (*i.e.*, ambiguines A–P). Also similar to the hapalindoles, both chlorinated and non-chlorinated (at C-13) variants generally showed comparable antimicrobial activity; however, unlike hapalindoles, there have to-date not been any isothiocyanate variants of the ambiguines reported. That said a multiple nitrile-containing ambiguines have been identified. In particular, ambiguine G nitrile was isolated from *F. ambigua*, and shown to have antibacterial and antifungal activity comparable to ambiguine isonitriles.

More recently, the fischambiguines were isolated, alongside several previously recognized ambiguines, from *F. ambigua* specifically based on antibacterial activity of extracts toward *Mycobacterium tuberculosis* and *Bacillus anthracis* [42]. Subsequently, purified fischambiguines A and B were found to be differentially active toward a range of bacteria at concentrations as low as 2 μM; in particular, fischambiguine B was generally more active (MIC ≥ 2–28 μM), and specifically inhibitory toward *M. tuberculosis* and *B. anthracis*, as well as *Staphylococcus aureus* and *M. smegmatis*, whereas fischambiguine A was moderately active against only *S. aureus* (MIC > 82 μM). It is proposed that presence of the epoxide in fischambiguine B might underlie the observed antibacterial activity of this variant, as the presence and absence of a chlorine atom (at C-13) has generally been found to have no significant effect on the antibacterial activity of the hapalindole-type alkaloids including the related ambiguines [37,38]).

Finally, antimicrobial activity has been also demonstrated for fischerindoles and welwitindolinones (Groups 7, 8 and 9). Fischerindole L was, when first isolated [43], found to be antifungal, and more recent studies [44] have, likewise, demonstrated inhibition of a range of bacteria and yeast by other variants. Similarly, the welwitindolinones—and particularly welwitindolinone A—were fungicidal [45]; however, members of the “welwitindolinone B” class (Group 9), on the other hand, have been exclusively linked to a reversal of multidrug resistance in human cancer cells (see Section 6.2, Biochemical, Molecular and Cellular Bioactivity).

### 6.2. Biochemical, Molecular and Cellular Bioactivity

Aside from their antimicrobial activity, subsequent studies have suggested a wide range of biological activity at the biochemical, molecular and cellular level that is, likewise, relevant to both the potential of the hapalindole-type alkaloids as drug leads, and to their possible role as toxins. At the cellular level, several of the indole alkaloids have been shown to be cytotoxic. In addition to antimicrobial activity, several of the hapalindoles (including A, C, E, H–J, U and X) were shown to be cytotoxic to both normal mammalian (*i.e.*, Vero) and various cancer cell lines [34,62]. Similar to antimicrobial activity, tetracyclic hapalindole A and J—and the unusual X—were particularly active in these studies, as was hapalindole H (although it was not comparatively tested for antimicrobial activity in the same study), whereas others (*i.e.*, I and U) were only moderately and/or variably cytotoxic [34]. The difference in cytotoxicity between, for example, hapalindole H and U is perhaps notable as they differ only in relative stereochemistry at C-10/11/15, and suggest a stereoselectivity of this biological activity. Similarly, in a prior study, the tricyclic variant, 12-*epi*-hapalindole E, was found to be moderately cytotoxic to a mouse myeloma cell line [62]. In addition, some oxidized hapalindoles (e.g., hapalonamide H, anhydrohapaloxindole A) shared cytotoxicity equivalent to the hapalindoles, whereas others (*i.e.*, 13-hydroxydechlorofontonamide) did not [34]. This finding is intriguing as both active and inactive variants (e.g., hapalonamide H and 13-hydroxydechlorofontonamide, respectively) include representatives which lack an intact indole ring.

Both tetra- and pentacyclic ambiguine isonitriles including numerous congeners (A–C, E, F, I, K–O), as well as the unique ambiguine G nitrile, were also found to be cytotoxic to a mammalian cell line [37,42], whereas ambiguine D isonitrile was shown to inhibit mitosis in plant cells [66]. In a recent study, it was specifically shown that ambiguine I isonitrile induced apoptosis in the MCF-7 breast cancer cell line, and led to arrest of cells in G1 phase [65]. Notably, the chemically related fischambiguines A and B were effectively not found to be cytoxotoxic (Mo *et al.*, 2010), although further investigation is clearly needed. Interestingly, however, given the considerable deviation in carbon backbone, the fischerindoles were found to be cytotoxic, and fischerindole L, in particular, was shown to be as equally cytotoxic (to both normal and cancer cells) as hapalindole-type alkaloids [44].

Arguably, the sub-class which has received the most attention in terms of cytotoxicity, particularly in relation to potential as anticancer drug, has been the welwitindolinones, and specifically welwitindolinone C isothiocyanate and its *N*-methyl derivative—or *welwistatin*—that were found to inhibit cell proliferation in various cancer cell lines, and moreover, to circumvent (as discussed below) multidrug resistance [67,68]. As a result of this compelling biological activity, considerable attention has been devoted, in particular, to synthetic studies of the welwistatin and other welwitindolinones [47].

**Figure 16:**
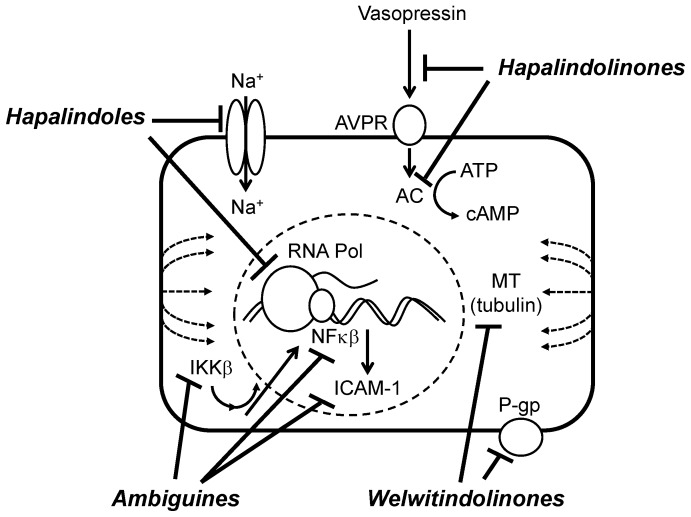
Overview of biochemical, molecular and cellular targets of the hapalindole-type alkaloids. Abbreviations: AC = adenylate cyclase; AVPR = arginine vasopressin receptor; ICAM-1 = interceullular adhesion molecule 1; IKKβ = inhibitor of nuclear factor kappa-B kinase subunit beta; MT = microtubulin; P-gp = p-glycoprotein; NFκB = nuclear factor kappa B; RNA pol = RNA polymerase.

With respect to biochemical or molecular underpinnings of the pharmacology of the hapalindole-type alkaloids, several possible mechanisms have, in fact, been identified (Figure 16). In a study of ambiguine I, for example, it was shown that observed induction of apoptosis and cell-cycle arrest could be specifically attributed to the inhibition of pathways involving the transcription factor, NF-κB, which has a well-established role in regulating multiple pathways involved in cancer progression including suppression of apoptosis [65]. It was generally shown in these studies that apoptosis occurred by way of a caspase-independent mechanism, and involved mitochondrial dysfunction including elevated levels of reactive oxygen species (ROS). Interestingly, studies of the antimitotic activity of ambiguine D in a plant cell model, likewise, demonstrated elevated ROS, and associated increases in lipid peroxidation, and was suggested to play a role in the observed cell death [66]. Moreover, immunoblotting studies of breast cancer cells treated with ambiguine I showed down-regulation of both p65 and p50 subunits of NF-κB, and the upstream kinase, IKKβ; in turn, ambiguine I treatment led to a down-regulation of intracellular cell adhesion protein, ICAM-1, which is involved in migration and invasion of cancer cells, suggesting a potential novel mechanism of the compound as an anticancer drug [65].

As discussed above, a considerable amount of attention has been placed on the welwitindolinones with respect to antiproliferative—and potential anticancer—activity. Although much of this research has focused on synthetic studies, the original characterization of the molecules and their biological activity identified compelling mechanistic details [67,68]. Specifically, welwitindolinones were isolated as part of studies focused on the identification of compounds which reverse MDR of cancer cells. Two variants, welwitindolinone C isothiocyanate and its *N-*methyl derivative, were specifically shown to abate acquired drug-resistance of a breast cancer cell line (*i.e.*, MCF-7/ADR) with respect to a diverse range of anticancer drugs, including taxol, vinblastine, colchicine, daunomycin and actinomycin D, at sub-micromolar concentrations [68]. In particular, *N*-methylwelwitindolinone C isothiocyanate showed pronounced activity (at concentrations below that of the described MDR-reversing drug, verapamil). And notably, the isonitrile variant of welwitindolinone C showed no activity, suggesting role of the isothiocyanate functional group. Similarly, accumulation of radiolabeled [^3^H]-taxol was observed in an MDR variant of an ovarian carcinoma cell line (*i.e.*, SK-VBL-1), and paralleled the potency of MDR-reversing potential of the variants in breast cancer cells; interestingly, however, the *N-*methyl derivative was not found to lead to the accumulation [^3^H]-taxol in treated cells, whereas the non-methylated welwitindolinone C isothiocyante and verapamil did. These studies, moreover, elucidate an apparent interaction with the P-glycoprotein (*i.e.*, ATP-binding efflux pump) pathway of MDR. Furthering the interest in these two compounds, a subsequent study demonstrated, in addition to MDR-reversing activity, antiproliferative activity of the welwistatins themselves, and specifically, interference with microtubule formation via inhibition of tubulin polymerization [67].

With respect to the hapalindoles, studies by Doan *et al.* [62,64] have pointed to inhibition of RNA polymerase as a possible mode of action. Investigating allelopathic potential, it was shown that 12-*epi*-hapalindole E inhibited the growth of a wide range of bacteria, fungi and algae, and more specifically, in a Gram-positive bacterial model (*i.e.*, *Bacillus subtilis* 168), inhibited both RNA and protein synthesis [62]. It was further demonstrated *in vitro* that the hapalindole inhibited RNA polymerase directly (rather than interacting with the DNA template). And subsequent studies using Gram-negative *E. coli* RNA polymerase both confirmed the direct interaction with the enzyme (and not DNA), and also demonstrated several notable difference from binding activity of other RNA polymerase inhibiting antibiotics, *i.e.*, rifampicin [64]. The study concluded, however, based on the relatively high concentration required for RNA polymerase inhibition, that additional mechanisms were likely involved in the antimicrobial activity of the hapalindoles.

Interestingly, several hapalindoles—including the tricyclic hapalindoles E and C isonitrile, and tetracyclic hapalindole L—were recently shown to modulate sodium channels in a neuroblastoma cell line [59]. The inhibition of veratridine-induced depolarization (as measure of sodium channels) occurred without cytotoxicity, and it is unlikely, therefore, to explain antimicrobial or cytotoxic activity. However, this mechanism was suggested to explain an observed insecticidal activity of these compounds [32,60] (see below). Moreover, this activity supports the potential of the hapalindoles as potential environmental toxins similar to other neurotoxic metabolites from cyanobacteria (and other “harmful algae”) including saxitoxins and anatoxin-a (see Section 8, Relevance as Harmful Algae).

In addition to the hapalindoles, oxidized congeners have also been investigated with respect to relevant pharmacological activity. Specifically, the hapalindolinones (*i.e.*, Group 3; Figure 3) were first identified in fact, as part of screening program to identify vasopressin antagonists [35]. Following isolation and chemical characterization, hapalindolinone A was shown to inhibit both binding of arginine vasopression to kidney cells, and also inhibit vasopressin stimulated adenylate cyclase. Accordingly, this compound has received attention as a possible drug lead for various associated diseases.

### 6.3. Toxicity to Plants and Animals

Finally, alongside pharmacological studies of the hapalindole-type alkaloids, several studies have investigated their toxic effects in multicellular organismal systems. In some cases, these effects could be attributed to biochemical, molecular or cellular mechanisms. For example, inhibition of mitosis by ambiguine D isonitrile was linked to phytotoxic effects, and specifically inhibition of seedling germination [66]; the phytotoxic activity was, in fact, successfully employed for bioassay-guided fractionation and purification of the compound. However, in most cases, the connection between mechanism and toxicity remains to be elucidated.

**Figure 17:**
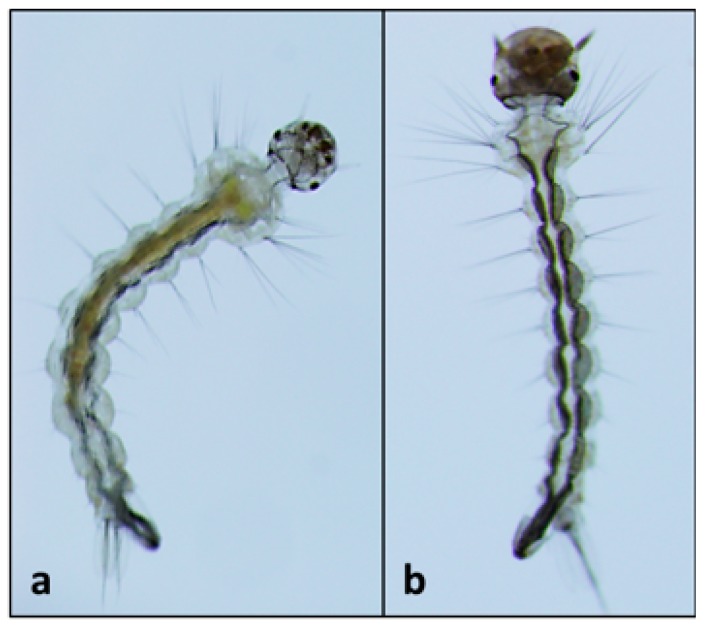
Inhibition of mosquito (*Aedes aegypti*) larval development by 12-*epi*-hapalindole H isonitrile. Shown are 2nd instar larvae treated with the alkaloid (**a**) showing development abnormalities compared to untreated control larvae (**b**). Most larvae treated with 12-epi-hapalindole H, however, do not transition from 1st to 2nd instar.

The hapalindole-type alkaloids have, for example, been shown as toxic to invertebrate animals, and particularly insects. The larvae of the fly, *Chironomus riparius*, was used as a model system to isolate several hapalindoles (*i.e.*, C, E, J and L including 12-epimers) as insecticidal agents from a biofilm-forming species of *Fischerella* [32,60]; in the same study, hapalindole J (identified as a new variant) was also found to be lethal to the freshwater crustacean, *Thamnocephalus platyurus*. Moreover, recent studies identified apparent sodium channel modulating activity of these same compounds, as discussed above, and it was proposed that this activity might explain the observed insecticidal activity [59]. In fact, other studies have, likewise, reported the apparent insecticidal activity of the hapalindole-type alkaloids. It was observed that welwitindolinones, and particularly *N*-methylwelwitindolinone C isothiocyanate, isolated as the major alkaloid from *W. intricate*, were insecticidal against blowfly larvae [45]. More recently an evaluation of crude extracts, and purified alkaloids, from a freshwater *Fischerella* isolate was shown to inhibit development of mosquito (*Aedes aegypti*) larvae [61]. Crude extracts, in this case, were lethal to larvae by day 4 of development, and larvae never transitioned beyond 1st instar stages. Both increased mortality and delays in development were, likewise, observed for larvae exposed to 12-*epi*-hapalindole H isonitrile compared to control (untreated) larvae; specifically, larvae exposed to the alkaloid generally were arrested at 1st instar stage after 5 days (whereas control larvae transitioned to 2nd instar by day 2), and development was observably impaired for larvae (only one) which did transition to 2nd instar (Figure 17).

**Figure 18:**
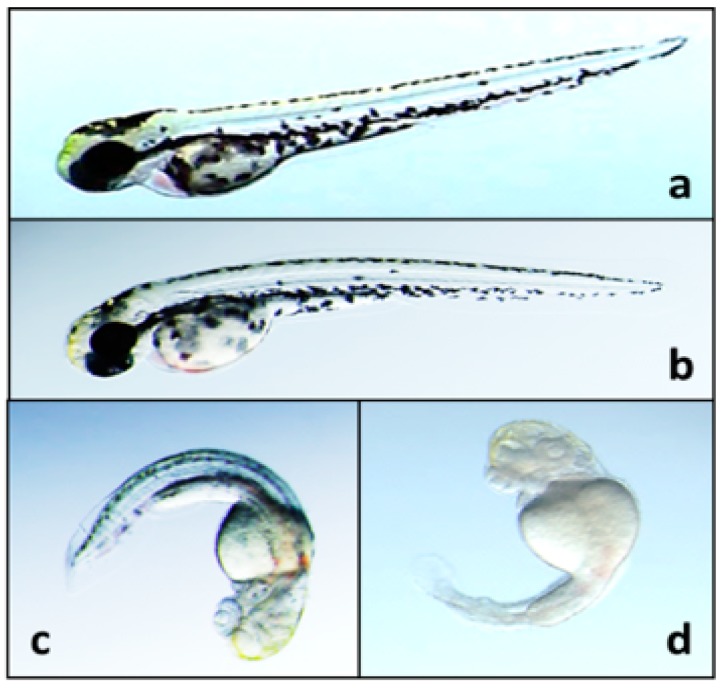
Teratogenicity of hapalindole-type alkaloids in the zebrafish (*Danio rerio*) embryo as a model of vertebrate development. Shown are control (untreated) embryos at 3 days post-fertilization (**a**); and embryos exposed for three days to 12-*epi*-ambiguine B nitrile at 10 μg/mL (**b**); and to 12-*epi*-hapalindole H isonitrile at 5 and 10 μg/mL (**c** and **d**, respectively) [61].

Most recently, the zebrafish (*Danio rerio*) embryo as a vertebrate model of teratogenicity (*i.e.*, development toxicity) was used to identify, purify and characterize several, diverse alkaloids from a *Fischerella* isolate [39,41,70]. Teratogenicity of the strain was first identified in screening studies, and initial characterization supported their identity as hapalindole-type alkaloids [70]. Subsequent studies were able to utilize bioassay-guided fractionation, based on observed teratogenicity, to purify and chemically characterize several variants including hapalindoles, ambiguines and fischerindoles, including new nitrile ambiguines [39,41]. Toxicity to embryos included—in addition to mortality at the highest exposure concentrations—impairment of melanophores, leading to lack of pigmentation, as well as deformity of the body axis and severe edemas (Figure 18). The observed impairment of melanophore development, in particular, is notable as melanocytes are derived from neural crest cells of embryos, may thereby support, as discussed below (Section 8, Relevance as Harmful Algae), a role of these compounds in myelinopathies observed among waterfowl exposed to epiphytic stigonematalean cyanobacteria [71]. The tetracyclic 12-*epi*-hapalindole H isonitrile was most closely linked to the developmental deformity observed in these studies, and was generally the most potent teratogen [39]. However, both ambiguine isonitrile and nitrile variants, as well as fischerindoles, purified in subsequent studies demonstrated observable teratogenicity albeit at higher concentrations, *i.e.*, ≥5–20 μM [41]. Generally speaking, these studies perhaps best support a potential contribution of the hapalindole-alkaloids to the toxicity of cyanobacteria from the order as a “harmful algae” in aquatic systems.

## 7. Possible Ecological Functions

A functional ecological role of the hapalindole-type alkaloids has been long suggested. Indeed, as discussed above, hapalindoles were first characterized from *Hapalosiphon* following observations of apparent allelopathy, and in particular, antialgal activity suggestive of a role in the inhibition of potentially competing aquatic microbes, *i.e.*, algae. More specifically, the inhibition of the growth of *Anabaena* by sympatric *H. intricatus* and *H. fontinalis* led to the isolation, and subsequent chemical characterization, of hapalindole A and B as the first archetypal variants of the class [29]. And several, subsequent studies pointed to the possible allelopathic role of the hapalindoles and ambiguines including antialgal activity [38,62,63].

Further supporting a role of the hapalindole-type alkaloids in allelopathy, studies by Gantar *et al.* [72] recently investigated numerous cyanobacterial and green algal isolates from the Florida Everglades, and associated South Florida waterways, by means of pairwise co-culturing experiments, and observed an isolate of *Fischerella* (52-1) as the most frequent inhibitor of sympatric algae. Moreover, extracts from *Fischerella* 52-1 were subsequently shown to inhibit photosynthesis, and disrupt thylakoid membranes, in *Chlamydomonas,* as a model green alga [72]. This same strain was subsequently shown to produce a diversity of indole alkaloids including hapalindoles, ambiguines and fischerindoles [39,41], and it was proposed that observed allelopathy may, therefore, be associated with these compounds; however, a definitive link between the observed allelopathy and these compounds has yet to be established. That said, hapalindoles and ambiguines were isolated from this same strain as major components of “exudates” produced by cultures [39], suggesting possible transport of these compounds to the extracellular environment as would be consistent with an allelopathic role.

In addition to the indole alkaloids, other bioactive metabolites from the order have been proposed to have a similar ecological role. Fischerellins A and B were shown to be only marginally antimicrobial (when tested alongside hapalindole alkaloids) against numerous species of bacteria and fungi [34]. However, the fisherellins were potently antialgal, inhibiting cyanobacteria and green-algae at nanomolar concentrations, despite having little or no effect on eubacteria. Indeed, this antialgal activity was used to isolate fischerellin A from *Fischerella* sp. [27,73]. Moreover, fischerellin A was subsequently shown to specifically inhibit photosystem II of algae and other photosynthetic organisms. (*i.e.*, plants) [27,74]. Similarly, although not directly investigated in this regard, the dendroamides isolated from *Stigonema* belong to the same class of cyclic peptides as—and bear, in fact, considerable structural similarity to—nostocyclamide from the cyanobacterial genus, *Nostoc*, which have been linked to allelopathy, and specifically inhibition of cyanobacteria, green-algae and diatoms [25,26].

Finally, in addition to the antimicrobial activity observed for hapalindole-type alkaloids, and potential relevance to allelopathy, several representatives have demonstrated insecticidal, and particularly insect larvicidal activity, as well as effects on freshwater zooplankton [32,45,60,61]. It is, therefore, tempting to speculate a possible role of these secondary metabolites against potential micrograzers. Such a role of cyanobacterial metabolites has, in fact, been generally proposed [75]. The observation of sodium channel modulation in neurons [59] by the hapalindoles—as an animal-specific mechanism of toxicity—further underscores a potential targeting of potential grazers, and most likely microinvertebrates (e.g., insect larvae, crustaceans). To date, however, no studies have directly investigated the anti-grazer potential of these compounds.

## 8. Relevance as Harmful Algae

As ubiquitous components of freshwater habitats, a link between production of *toxic* metabolites by the stigonematalean cyanobacteria, and possible environmental human health concerns, is proposed. In terms of potential toxicity of the indole alkaloids from Stigonematales there have, however, been rather limited documented links to negative impacts on human, animal or ecocystem health in this regard. That said, recognized cyanobacterial toxins, and specifically the microcystins [11,12,13] and BMAA [14] have been identified from members of the order (see Section 3, Stigonematales as a Source of Bioactive Metabolites). Moreover, a preponderance of toxicological studies of the indole alkaloids effectively supports the potential of these metabolites as naturally occurring toxins.

Perhaps most relevant, in this regard, is the recent identification of teratogenicity of the indole alkaloids from a freshwater *Fischerella* strain, specifically using the zebrafish (*Danio rerio*) embryo model of vertebrate development [39,41,70]. In these studies, developmental toxicity was observed in zebrafish embryos ambiently exposed to the alkaloids, suggesting the potential from direct exposure in the environment (*i.e.*, the water column). And, as a freshwater teleost fish model, these findings would directly support a potential effect of these teratogens on fish populations in these habitats. Moreover, as a vertebrate model, it is, of course, possible that these observations could be extrapolated to other vertebrates including humans.

Alongside observed vertebrate toxicity, the recently reported effects of hapalindoles on sodium channels of neurons, likewise, indirectly supports potential neurotoxicity of these compounds, and possible concerns, therefore, as toxigenic algae in aquatic systems [59]. Indeed, the authors of these studies compared activity of the hapalindoles, in this regard, to the known sodium-channel blocker, and recognized HAB toxin, neosaxitoxin, and suggested a common mechanism. It was accordingly proposed that hapalindoles should be considered alongside other neurotoxic cyanotoxins (e.g., saxitoxins, anatoxin-a).

Although there have been no direct links between characterized bioactive (*i.e.*, potentially toxic) metabolites from the Stigonematales, and either human or animal intoxiciations, there has been indirect evidence in this regard. Most notably, on-going studies have documented a link between apparent metabolites of a stigonematalean cyanobacterial species, and well documented *avian ventricular myelinopathy* (AVM) among various bird species including waterfowl (e.g., American coots, various duck species), and other birds (e.g., bald eagles). Numerous studies, in this regard, have documented AVM as a neurological disease among birds, and feeding studies have linked the disorder to consumption of aquatic vegetation, and specifically the invasive *Hydrilla verticillata* [76]. More recently, however, subsequent studies identified, specifically using molecular techniques, an apparent cyanobacterial epiphyte of *H. verticillata,* and correlated presence to the occurrence of AVM [77]. And most recently, these studies have characterized a new species, *Aetokthonos hydrillicola*, specifically within the Stigonematales, as the epiphytic cyanobacterium [10]. Feeding studies with the plant material containing this epiphytic cyanobacterium, as well as extracts prepared from this material, generally confirm a link between AVM and a cyanobacterial metabolite [71,78]. To date, however, the actual toxins involved in AVM have yet to be identified, and work is currently in progress to do so (S. Wilde, personal communication).

That said, hapalindole-type alkaloids represent clear candidates as toxins associated with AVM. It was, in fact, recently noted by Cagide *et al.* [59] that neurotoxicity, and apparent sodium channel blocking, of hapalindole could contribute to this neurological disorder. Likewise, the observed teratogenicity of the alkaloids [39,70] might suggest a mechanism. Specifically, the teratogenic alkaloids from *Fischerella* seemingly affected neural crest-derived cells (*i.e.*, melanophores) in the zebrafish embryo model [39]. Glia, as the producers of myelin in the nervous system, are, likewise, derived from neural crest; and the vacuolization of myelin (*i.e.*, “white matter”) in the brains of AVM-affected birds might be consistent with apparently cell-lineage specific activity of the teratogenic indole alkaloids. In addition, a recent study identified the neurotoxic non-proteinogenic amino acid, β-methylamino-l-alanine (BMAA), from this epiphyte [14], and suggested a possible role in AVM. Clearly, identification of the toxic principles involved in AMV will be key to establishing a link to any of these metabolites, or others.

## 9. Potential Source of Drug Leads

Finally, in terms of the potential of these indole alkaloids as leads to pharmaceutical, the literature is, in fact, rather limited. However, pharmacological data—along with continued synthetic interest in these molecules—do support potential future development in this regard. As detailed above, cellular and molecular targets identified for the hapalindole-type alkaloids include appreciable antibiotic activity, antiproliferative effects against diverse cancer cells, reversal of MDR, selective inhibition of RNA polymerases and antagonism of vasopressin. Underscoring this pharmacological potential it has been regularly noted that antibiotic and antimycotic activity of the hapalindoles and ambiguines are comparable to established drugs in this regard; for example, hapalindole T inhibited *M. tuberculosis* as potently as the established anti-tuberculosis drug, rifampicin, and ambiguines were comparable with commercially available antibiotics (e.g., streptomycin) and antifungal drugs (e.g., puramycin, amphotericin B) [31,38]. On the other hand, the welwistatins (*i.e.*, welwitindolinones) have been perhaps most actively studied as compelling anticancer drug candidates due to a combined antimitotic activity, and MDR-reversing activity which is comparable with established drugs (e.g., veraprimil) in this regard [67,68]. Accordingly, to-date there have been no less than twenty-three published synthetic studies targeting this latter sub-class of alkaloids [47].

Consistent with pharmacological potential, several patents have been filed for a diverse range of metabolites from the Stigonematales. Numerous patents have been filed in relation to the reported biological activity of the hapalindoles and ambiguines including, in particular, antifungal, antibacterial and antitumor activity, as well as to methods for their production including biosynthesis from cultures. Similarly, patents regarding the anticancer potential of welwistatin, and vasopressin antagonist activity of the hapalindolinones, specifically as a treatment for congestive heart failure, hypertension, edema and hyponatremia, have been filed within the past 20 years. The hapalindole alkaloids are clearly poised with respect to future development as drug leads. To date, however, there have been no clinical studies of the hapalindole-type alkaloids, and future studies in this regard are obviously needed, along with continued pharmacological characterization, and supporting chemical synthesis (and, alternatively, biosynthetic studies), to realize this potential.

## 10. Conclusions

Taken together, the existing data regarding the bioactive secondary metabolites from the Stigonematales paint a burgeoning picture of the role of these compounds—and particularly the hapalindole-type alkaloids—with respect to human and environmental health. Continued chemical and pharmacological/toxicological characterization of these metabolites, and rapidly evolving insight as to their biosynthetic origins, reveals the impressive depth of this intriguing metabolic class of compounds. However, realization of the potential of these compounds as drugs (for an apparently wide range of important biomedical targets), as well as a suggested role of these metabolites with respect to both ecology of the order, and as a novel class of environmental toxins, remains an exciting area for future scientific exploration. In terms of ecological roles of these compounds, much remains to be studied. Likewise, identification (and clarification) of links between bioactive metabolites from the stigonematalean cyanobacteria and intoxications will be key to confirming a role in environmental health (*i.e.*, as HABs). Finally, while a preponderance of pre-clinical data support the potential of these compounds as drugs, clinical studies are, in particular, needed. The current state of the science with respect to these widespread cyanobacteria clearly support future studies with respect to all of these aspects.

## Figures and Tables

**Table 1:** Summary of the reported biological activity of hapalindole-type alkaloids from Stigonematales including inhibition of microbes, cytotoxicity and plant/animal toxicity, as well as biochemical, molecular and cellular targets.

	Antimicrobial			Cytotoxicity	Biochemical, Molecular or Cellular	Plant/Animal Toxicity
	Bacteria	Fungi	Algae			
**Hapalindoles**						
Tetracyclic (*Group 1*)	A, G–H, I–J, T, X [29,31,34,42]	G [42]	A, J, X [34]	A, H–J, U, X [34]	Modulate Na^+^ channels in neuroblastoma [59]	Insecticidal [32,60,61] Teratogenicity/vertebrate toxicity [39,41]
Tricyclic (*Group 2*)	E [62]	E [62]	E–F [62,63]	C, E [34,62]	Modulate Na^+^ channels in neuroblastoma [59] RNA polymerase inhibition [62,64]	Insecticidal [32,60]
Hapalindolinones (*Group 3*)					Inhibits arginine vassopressin binding, and adenylate cyclase [35]	
**Ambiguines**						
Tetracyclic (*Group 4*)	A–C, H [38,42]	A–C, H [38,42]		A–C [42]		Teratogenicity/vertebrate toxicity [39,41]
Pentacyclic (*Group 5*)	E–F, G nitrile, I, K–O [37,38,42]	E–F, I, K–O, P [37,38,42]		E–F, G nitrile, K–O [37,42]	Apoptosis, inhibition of NF-kb, IKKb and ICAM-1 [65] Inhibits mitosis (plant cells) [66] Elevates ROS [65,66]	Phytotoxic [66]
Fischambiguines (*Group 6*)	A, B [42]	A [42]				
**Fischerindoles** (*Group 7*)	L [34]	L [34]		descholor I nitrile, L [34,44]		Teratogenicity/vertebrate toxicity [41]
**Welwitindolinones**						
Welwitindolinone A (*Group 8*)		A [45]				
Welwitindolinone B (*Group 9*)				*N*-methyl C isothiocyanate [67]	Inhibits microtubules and mitosis [67] Inhibit P-glycoprotein MDR [67,68]	Insecticidal [45]

## References

[1] Singh R.K., Tiwari S.P., Rai A.K., Mohapatra T.M. (2011). Cyanobacteria: An emerging source for drug discovery. J. Antibiot..

[2] Dixit R.B., Suseela M.R. (2013). Cyanobacteria: Potential candidates for drug discovery. Antonie Van Leeuwenhoek.

[3] Zanchett G., Oliveira-Filho E.C. (2013). Cyanobacteria and cyanotoxins: From impacts on aquatic ecosystems and human health to anticarcinogenic effects. Toxins.

[4] Dittmann E., Gugger M., Sivonen K., Fewer D.P. (2015). Natural product biosynthetic diversity and comparative genomics of the cyanobacteria. Trends Microbiol..

[5] Ibelings B.W., Backer L.C., Kardinaal W.E., Chorus I. (2015). Current approaches to cyanotoxin risk assessment and risk management around the globe. Harmful Algae.

[6] Wilmotte A., Herdman M., Boone D.R., Castenholz R.W. (2001). Phylogenetic relationships among the cyanobacteria based on 16s rRNA sequences. Bergey’s Manual of Systematic Bacteriology.

[7] Anagnostidis K., Komárek J. (1990). Modern approach to the classification system of Cyanophytes 5-Stigonematales. Arch. Hydrobiol. Suppl..

[8] Gugger M.F., Hoffmann L. (2004). Polyphyly of true branching cyanobacteria (Stigonematales). Int. J. Sytemat. Evol. Microbiol..

[9] Dagan T., Roettger M., Stucken K., Landan G., Koch R., Major P., Gould S.B., Goremykin V.V., Rippka R., Tandeau de Marsac N. (2013). Genomes of Stigonematalean cyanobacteria (Subsection V) and the evolution of oxygenic photosynthesis from prokaryote to plastids. Genome Biol. Evol..

[10] Wilde S.B., Johansen J.R., Wilde H.D., Jiang P., Bartelme B.A., Haynie R.S. (2014). *Aetokthenos hydrillicola gen. et* sp. *nov.:* Epiphytic cyanobacteria on invasive aquatic plants implicated in Avian Vacuolar Myelinopathy. Phytotaxa.

[11] Fiore M.F., Genuária D.B., da Silva C.S., Shishido T.K., Moraes L.A., Cantúsio N.R., Silva-Stenico M.E. (2009). Microcystin production by a freshwater spring cyanobacterium of the genus *Fischerella*. Toxicon.

[12] Prinsep M.R., Caplan F.R., Moore R.E., Patterson G.M.L., Honkanen R.E., Boynton A.L. (1992). Microcystin-LA from a blue-green algae belonging to the Stigonematales. Phytochemistry.

[13] Cirés R., Alvarez-Roa C., Wood S.A., Puddick J., Loza V., Heiman K. (2014). First report of microcystins producing *Fischerella* sp. (Stigonematales, Cyanobacteria) in tropical Australia. Toxicon.

[14] Bidigare R.R., Christensen S.J., Wilde S.B., Banack S.A. (2009). Cyanobacteria and BMAA: Possible linkage with avian vacuolar myelinopathy (AVM) in the southeastern United States. Amyotroph. Lateral Scler..

[15] Niiyama Y., Tuji A., Tsujimura S. (2011). *Umezakia natans* does not belong to the Stigonemataceae to Nostocaceae. Fottea.

[16] Falch B.S., König G.M., Wright A.D., Sticher O., Angerhofer C.K., Pezzuto J.M., Bachmann H. (1995). Biological activities of cyanobacteria: Evaluation of extracts and pure compounds. Planta Med..

[17] Wright A.D., Papendorf O., König G.M. (2005). Ambigol C and 2,4-dichlorobenzoic acid, natural products produced by the terrestrial cyanobacterium *Fischerella ambigua*. J. Nat. Prod..

[18] Hilliwig M.L., Liu X. (2014). A new family of iron-dependent halogenases acts on freestanding substrates. Nat. Chem. Biol..

[19] Gloaguen V., Morvan H., Hoffmann L., Catherine O.S., Kraemer M., Krausz P. (2007). Bioactive capsular polysaccharide from the thermophilic cyanophyte/cyanobacterium *Mastigocladus laminosus*—Cytotoxic properties. Planta Med..

[20] Prinsep M.R., Moore R.E., Levine I.A., Patterson G.M. (1992). Westiellamide, a bistratamide-related cyclic peptide from the blue-green alga *Westelliopsis prolifica*. J. Nat. Prod..

[21] Stratmann K., Burgoyne D.L., Moore R.E., Patterson G.M.L., Smith C.D. (1994). Hapalosin, a cyanobacterial cyclic depsipeptide with multidrug-resistance reversing activity. J. Org. Chem..

[22] Kang H.S., Krunic A., Orjala J. (2012). Stigonemapeptin, an Ahp-containing depsipeptide with elastase inhibitory activity from the bloom-forming freshwater cyanobacterium *Stigonema* sp.. J. Nat. Prod..

[23] Ogino J., Moore R.E., Patterson G.M., Smith C.D. (1995). Dendroamides, new cyclic hexapeptides from a blue-green alga. Multidrug resistance reversing activity of dendroamide A. J. Nat. Prod..

[24] Foster M.P., Concepcion G.P., Caraan G.B., Ireland C.M. (1992). Bistratamides C and D. Two new oxazole-containing cyclic hexapeptides isolated from a Philippine *Lissoclinum bistratum* ascidian. J. Org. Chem..

[25] Todorova A.K., Jüttner F. (1995). Nostocyclamide: A new macrocyclic, thiazole-containing allelochemical from *Nostoc* sp. 31. J. Org. Chem..

[26] Jüttner F., Todorova A.K., Walch N., von Philipsborn W. (2001). Nostocyclamide M: A cyanobacterial cyclic peptide with allelopathic activity from *Nostoc* 31. Phytochemistry.

[27] Hagmann L., Jüttner F. (1996). Fischerellin A, a novel photosystem-II inhibiting allelochemical of the cyanobacterium, *Fischerella muscicola*, with antifungal and herbicidal activity. Tetrahedron Lett..

[28] Papke U., Gross E.M., Francke W. (1997). Isolation, identification and determination of the absolute configuration of fischerellin B. A new algicide from the freshwater cyanobacterium *Fischerella muscicola*. Tetrahedron Lett..

[29] Moore R.E., Cheuk C., Patterson G.M.L. (1984). Hapalindoles: New alkaloids from the blue-green alga *Hapalosiphon fontinalis*. J. Am. Chem. Soc..

[30] Moore R.E., Cheuk C., Yang X.-Q.G., Patterson G.M.L., Bonjouklian R., Smitka T., Mynderse J.S., Foster R.S., Jones N.D., Swartzendruber J.K. (1987). Hapalindoles, antibacterial and antimycotic alkaloids from the cyanophyte *Hapalosiphon fontinalis*. J. Org. Chem..

[31] Asthana R.K., Srivastava A., Singh A.P., Deepali, Singh S.P., Nath G., Srivastava R., Srivastava B.S. (2006). Identification of an antimicrobial entity from the cyanobacterium *Fischerella* sp. isolated from the bark of *Azadirachta indica* (Neem) tree. J. Appl. Phycol..

[32] Becher P.G., Keller S., Jung G., Süssmuth R.D., Jüttner F. (2007). Insecticidal activity of 12-*epi*-hapalindole J isonitrile. Phytochemistry.

[33] Moore R.E., Yang X.-Q.G., Patterson G.M.L., Bonjouklian R., Smitka T.A. (1989). Hapalonamides and other oxidized hapalindoles from *Hapalosiphon fontinalis*. Phytochemistry.

[34] Kim H., Lantvit D., Hwang C.H., Kroll D.J., Swanson S.M., Franzblau S.G., Orjala J. (2012). Indole alkaloids from two cultured cyanobacteria, *Westelliopsis* sp. and *Fischerella muscicola*. Bioorg. Med. Chem..

[35] Schwartz R.E., Hirsch C.F., Spring J.P., Pettibone J., Zink D.L. (1987). Unusual cyclopropane-containing hapalindolinones from a cultured cyanobacterium. J. Org. Chem..

[36] Smitka T.A., Bonjouklian R., Doolin L., Jones N.D., Deeter J.B., Yoshida W.Y., Prinsep M.R., Moore R.E., Patterson G.M.L. (1992). Ambiguine isonitriles, fungicidal hapalindole-type alkaloids from three genera of blue-green algae belonging to the Stigonemataceae. J. Org. Chem..

[37] Mo S., Krunic A., Chlipala G., Orjala J. (2009). Antimicrobial ambiguine isonitriles from the cyanobacterium *Fischerella ambigua*. J. Nat. Prod..

[38] Raveh A., Carmeli S. (2007). Antimicrobial ambiguines from the cyanobacterium *Fischerella* sp. collected in Israel. J. Nat. Prod..

[39] Walton K., Gantar M., Gibbs P.D., Schmale M.C., Berry J.P. (2014). Indole alkaloids from *Fischerella* inhibit vertebrate development in the zebrafish (*Danio rerio*) embryo model. Toxins.

[40] Huber U., Moore R.E., Patterson G.M.L. (1998). Isolation of a nitrile-containing indole alkaloid from the terrestrial blue-green alga *Hapalosiphon delicatulus*. J. Nat. Prod..

[41] Steele D., Berry J.P. Isolation, chemical characterization and comparative toxicology of structurally diverse indole alkaloids from *Fischerella* as teratogens. Mar. Drugs.

[42] Mo S., Krunic A., Santarsiero B.D., Franzblau S.G., Orjala J. (2010). Hapalindole-related alkaloids from the cultured cyanobacterium *Fischerella ambigua*. Phytochemistry.

[43] Park A., Moore R.E., Patterson G.M.L. (1992). Fischerindole L, a new isonitrile from the terrestrial blue-green alga *Fischerella muscicola*. Tetrahedron Lett..

[44] Kim H., Krunic A., Lantvit D., Shen Q., Kroll D.J., Swanson S.M., Orjala J. (2012). Nitrile-containing fischerindoles from the cultured cyanobacterium *Fischerella* sp.. Tetrahedron.

[45] Stratmann K., Moore R.E., Bonjouklian R., Deeter J.B., Patterson G.M.L., Shaffer S., Smith C.D., Smitka T.A. (1994). Welwitindolinones, unusual alkaloids from the blue-green algae *Hapalosiphon welwitschii* and *Westiella intricate:* Relationship to the fischerindoles and hapalindoles. J. Am. Chem. Soc..

[46] Wood J.L. (2012). Total synthesis: Welwitindolinone is well worth it. Nat. Chem..

[47] Bhat V., Dave A., MacKay J.A., Rawal V.H. (2014). The chemistry of hapalindoles, fischerindoles, ambiguines and welwitindolinones. Alkaloids Chem. Biol..

[48] Jimenez J.I., Huber U., Moore R.E., Patterson G.M. (1999). Oxidized welwitindolinones from terrestrial *Fischerella* sp.. J. Nat. Prod..

[49] Hilliwig M.L., Fuhrman H.A., Ittiiamornkul K., Sevco T.J., Kwak D.H., Liu X. (2014). Identification and characterization of a welwitindolinone alkaloid biosynthetic gene cluster in the Stigonematalean cyanobacterium *Hapalosiphon welwitschii*. ChemBioChem.

[50] Knowles C.J. (1976). Microorganisms and cyanide. Bacteriol. Rev..

[51] Pistorius E.K., Voss H. (1980). Some properties of a basic l-amino acid oxidase from *Anacystis nidulans*. Biochim. Biophys. Acta.

[52] Bornemann V., Patterson G.M.L., Moore R.E. (1988). Isonitrile biosynthesis in the cyanophyte *Hapalosiphon fontinalis*. J. Am. Chem. Soc..

[53] Garson M.J., Simpson J.S. (2004). Marine isocyanides and related natural products—Structure, biosynthesis and ecology. Nat. Prod. Rep..

[54] Hilliwig M.L., Zhu Q., Liu X. (2014). Biosynthesis of ambiguine indole alkaloids in cyanobacterium *Fischerella ambigua*. ACS Chem. Biol..

[55] Liu X., Hilliwig M.L., Koharudin L.M., Gronenborn A.M. (2016). Unified biogenesis of ambiguine, fischerindole, hapalindole and welwitindolinone: Identification of a monogeranylated indolenine as a cryptic common biosynthetic intermediate by an unusual magnesium-dependent aromatic prenyltransferase. Chem. Commun..

[56] Richter J.M., Ishihara Y., Masuda T., Whitefield B.W., Llamas T., Pohjakallio A., Baran P.S. (2008). Enantiospecific total synthesis of the hapalindoles, fischerindoles and welwitindolinones via a redox economic approach. J. Am. Chem. Soc..

[57] Micallef M.L., Sharma D., Bunn B.M., Gerwick L., Viswanathan R., Moffitt M.C. (2014). Comparative analysis of hapalindole, ambiguine and welwitindolinone gene clusters and reconstitution of indole-isonitrile biosysynthesis from cyanobacteria. BMC Microbiol..

[58] Li S., Lowell A.N., Yu F., Raveh A., Newmister S.A., Bair N., Schaub J.M., Williams R.M., Sherman D.H. (2015). Hapalindole/ambiguine biogenesis is mediated by a Cope Rearrangement, C-C bond-forming cascade. J. Am. Chem. Soc..

[59] Cagide E., Becher P.G., Louzao M.C., Espiña B., Vieytes M.R., Jüttner F., Botana L.M. (2014). Hapalindoles from the cyanobacterium *Fischerella*: Potential sodium channel modulators. Chem. Res. Toxicol..

[60] Becher P.G., Jüttner F. (2005). Insecticidal compounds of the biofilm-forming cyanobacterium *Fischerella* sp. (ATCC 43239). Environ. Toxicol..

[61] Walton K.E. (2012). Identification, Isolation and Characterization of Developmental Toxins from the Cyanobacterium *Fischerella* 52–1 Using the Zebrafish (*Danio rerio*) Embryo Model. M.S. Thesis.

[62] Doan N.T., Rickards R.W., Rotschild J.M., Smith G.D. (2000). Allelopathic actions of the alkaloids 12-*epi*-hapalindole E isonitrile and calothrixin A from cyanobacteria of the genera *Fischerella* and *Calothrix*. J. Appl. Phycol..

[63] Etchagaray A., Rabello E., Dieckmann R., Moon D.H., Fiore M.F., von Döhren H., Tsai S.M., Neilan B.A. (2004). Algicide production by the filamentous cyanobacterium *Fischerella* sp. CENA 19. J. Appl. Phycol..

[64] Doan N.T., Stewart P.R., Smith G.D. (2001). Inhibition of bacterial RNA polymerase by the cyanobacterial metabolite 12-epi-hapalindole E isonitrile and calothrixin A. FEMS Microbiol. Lett..

[65] Acuña U.M., Zi J., Orjala J., Carcache de Blanco E.J. (2015). Ambiguine I isonitrile from *Fischerella ambigua* induces caspase-independent cell death in MCF-7 hormone dependent breast cancer cells. Int. J. Cancer Res..

[66] Koodkaew I., Sunohara Y., Matsuyama S., Matsumoto H. (2012). Isolation of ambiguine D isonitrile from *Hapalosiphon* sp. and characterization of its phytotoxic activity. Plant Growth Regul..

[67] Zhang X., Smith C.D. (1996). Microtubule effects of welwistatin, a cyanobacterial indolinone that circumvents multiple drug resistance. Mol. Pharmacol..

[68] Smith C.D., Zilfou J.T., Stratmann K., Patterson G.M., Moore R.E. (1995). Welwitindolinone analogues that reverse P-glycoprotein-mediated multiple drug resistance. Mol. Pharmacol..

[69] Klein D., Daloze D., Braekman J.C., Hoffmann L., Demoulin V. (1995). New hapalindoles from the cyanophyte *Hapalosiphon laingii*. J. Nat. Prod..

[70] Berry J.P., Gantar M., Gibbs P.D.L., Schmale M.C. (2007). The zebrafish (*Danio rerio*) embryo as a model system for identification and characterization of developmental toxins from marine and freshwater microalgae. Comp. Biochem. Physiol. C Toxicol. Pharmacol..

[71] Wiley F.E., Wilde S.B., Birrenkott A.H., Williams S.K., Murphy T.M., Hope C.P., Bowerman W.W., Fischer J.R. (2007). Investigation of the link between avian vacuolar myelinopathy and a novel species of cyanbacteria through laboratory feeding trails. J. Wildl. Dis..

[72] Gantar M., Berry J.P., Thomas S., Wang M., Perez R., Rein K.S. (2008). Allelopathic activity among cyanobacteria and microalgae isolated from Florida freshwater habitats. FEMS Microbiol. Ecol..

[73] Gross E.M., Wolk C.P., Jüttner F. (1991). Fischerellin, a new allelochemical from the freshwater cyanobacterium *Fischerella muscicola*. J. Phycol..

[74] Srivastava A., Jüttner F., Strasser R.J. (1998). Action of the allelochemical, fischerellin A, on photosystem II. Biochim. Biophys. Acta Bioenerg..

[75] Berry J.P., Gantar M., Perez M.H., Berry G., Noriega F. (2008). Cyanobacterial toxins as allelochemicals with potential applications as algaecides, herbicides and insecticides. Mar. Drugs.

[76] Birrenkott A.H., Wilde S.B., Hains J.J., Fischer J.R., Murphy T.M., Hope C.P., Parnell P.G., Bowermann W.W. (2004). Establishing a food-chain link between aquatic plant material and avian vacuolar myelinopathy in mallards (*Anas platyrhynchos*). J. Wildlif. Dis..

[77] Wilde S.B., Murphy T.M., Hope C.P., Habrun S.K., Kempton J., Birrenkott A., Wiley F., Bowermann W.W., Lewitus A.J. (2005). Avian vacuolar myelinopathy linked to exotic aquatic plants and novel cyanobacterial species. Environ. Toxicol..

[78] Wiley F.E., Twiner M.J., Leighfield T.A., Wilde S.B., Van Dolah F.M., Fischer J.R., Bowerman W.W. (2009). An extract of *Hydrilla verticillata* and associated epiphytes induces avian vacuolar myelinopathy in laboratory mallards. Environ. Toxicol..

